# Analysis of FLA Gene Family in Four Poaceae Species and Functional Characterization of *SiFLA6* in Saline–Alkali Tolerance

**DOI:** 10.3390/plants15213260

**Published:** 2026-10-22

**Authors:** Yun Li, Yanan Yang, Fuxing Xiang, Chengyu Li, Cuili Hao, Xiaohu Lin, Yucui Han, Yu Zhao

**Affiliations:** 1 College of Agronomy and Biotechnology, Hebei Normal University of Science and Technology/Hebei Key Laboratory of Crop Stress Biology, Qinhuangdao 066000, China; 2 Chengde Academy of Agriculture and Forestry Sciences, Chengde 067000, China; 3 Institute of Millet Crops, Hebei Academy of Agriculture and Forestry Sciences/Key Laboratory of Genetic Improvement and Utilization for Featured Coarse Cereals (Co-Construction by Ministry and Province), Ministry of Agriculture and Rural Affairs/The Key Research Laboratory of Minor Cereal Crops of Hebei Province, Shijiazhuang 050035, China

**Keywords:** foxtail millet, green foxtail, switchgrass, sorghum, *FLA* gene family, *SiFLA6*, saline–alkali stress

## Abstract

Fasciclin-like arabinogalactan proteins (FLAs) are ubiquitous in the plant kingdom and play crucial roles throughout the entire life cycle of plant growth and development. To gain valuable genetic insights and to systematically dissect the biological functions of *FLAs*, we performed a comprehensive genome-wide analysis of the *FLA* gene family in four Poaceae species, including foxtail millet, green foxtail, switchgrass, and sorghum, along with functional characterization of key candidate genes. A total of 121 *FLA* family members were identified across the four species. Phylogenetic tree analysis classified these *FLAs* into four distinct subfamilies, within which the genes exhibited highly conserved gene structures and conserved motifs. The promoters of most *FLA* genes contained abundant *cis*-acting elements associated with stress and hormone responses. Furthermore, the saline–alkali tolerance-associated candidate gene *SiFLA6* was cloned, and T_3_-generation homozygous overexpression lines (OE-1 and OE-2) were generated via genetic transformation. Subcellular localization testing using the OE-1 line confirmed that SiFLA6 is located in the endoplasmic reticulum. Phenotypic assays showed that root growth of the OE-1 and OE-2 lines was less inhibited under saline–alkali stress than that of wild-type *Arabidopsis thaliana* (WT). Consistent with the phenotypic changes, the OE-1 line exhibited significantly higher shoot fresh and dry biomass, chlorophyll content, and activities of SOD, CAT, and POD, as well as higher K^+^/Na^+^ and Ca^2+^/Na^+^ ratios compared with WT plants. In contrast, the accumulation of H_2_O_2_ and MDA in the overexpression line was markedly reduced. Collectively, these results indicate that *SiFLA6* overexpression improves plant saline–alkali tolerance.

## 1. Introduction

Arabinogalactan proteins (AGPs) are a class of glycoproteins rich in hydroxyproline (Hyp). Being high glycosylation, AGPs are widely distributed in plant cell walls and plasma membranes and play critical roles in plant growth and development [1]. Based on differences in the core protein backbones, AGPs are classified into seven categories: classical AGPs, non-classical AGPs, lysine-rich AGPs, AG peptides, fasciclin-like arabinogalactan proteins (*FLA*s), xylogen-like AGPs (XLAs), and phytocyanin-like AGPs (PLAs) [2,3]. AGPs are primarily composed of repetitive sequence regions formed by proline (Pro), alanine (Ala), serine (Ser), and threonine (Thr), which are defined as PAST sequences. Classical AGPs contain more than 50% PAST sequences, whereas *FLA*s generally contain a PAST sequence proportion of less than 35% [4,5]. In addition to the AGP glycosylation region, *FLA*s also contain fasciclin (FAS) domains [6]. The FAS domain consists of 110–150 amino acids. Although its overall sequence similarity is low among different proteins, all FAS domains contain highly conserved the H1 and H2 motifs of approximately 10 amino acids. Proteins containing FAS domains have been confirmed to be involved in cell adhesion [7,8]. Most *FLA* genes encode proteins with an N-terminal signal peptide (SP) and a C-terminal glycosylphosphatidylinositol (GPI) anchor, and these proteins are mainly localized on the cell surface [9,10].

*FLAs* serve crucial regulatory functions throughout all stages of plant growth and development [11]. In *Arabidopsis*, *AtFLA1* and *AtFLA2* facilitate the initiation and development of adventitious roots in vitro [12]; *AtFLA3* is required for male and female gametophyte development [13]; *AtFLA4* modulates root growth through the abscisic acid (ABA) signaling pathway [8]; and the double knockout line of *AtFLA11* and *AtFLA12* alters the secondary cell wall composition of stems and reduces their tensile strength [14]. Overexpression of *GhFLA1* in cotton markedly promotes fiber elongation and remodels cell wall components [15]. *DEAP1*, which encodes a rice FLA protein, is involved in male reproductive development [16]. Since the FLA gene family is of vital importance to the growth and development of plants, it can respond to various abiotic stresses, including salinity [17], cold [18], drought [19], and heat [20]. To date, genome-wide identification of the *FLA* gene family has been performed in numerous plant species, revealing 21, 24, 34, 30, and 23 members in *Arabidopsis* [8], rice [3], wheat [20], banana [21], and hemp [9], respectively.

Salt and alkali stress is one of the main abiotic stresses that plants encounter. Neutral salt stress is caused by NaCl, Na_2_SO_4,_ and other neutral salts. These ions (Na^+^ and Cl^−^) interfere with various metabolic functions of the plant, thereby significantly affecting its tolerance [22]. Alkaline stress is caused by NaHCO_3_ and Na_2_CO_3_, and these substances further increase the pH value under salt stress conditions. The high pH values also severely disrupt the pH stability of cells, damage the integrity of cell membranes, and affect crop growth [23]. Saline–alkali land, due to its high salt content and high pH, has a severe inhibitory effect on the growth and development of most plants, resulting in a significant decline in crop yields and even crop failure [24]. Currently, approximately 1.381 × 10^9^ ha hectares of land worldwide are affected by salinization, accounting for 10.7% of the Earth’s land area [25]. Selecting new crop varieties with strong saline–alkali tolerance is an important and economically effective way to utilize saline–alkali land. Analyzing the FLA regulatory mechanism of crop saline–alkali tolerance is of great significance for improving the saline–alkali tolerance of crops.

Foxtail millet (*Setaria italica*), an important cereal crop in the Poaceae family, was domesticated from its wild progenitor green foxtail (*Setaria viridis*) over 8700 years ago [26]. Foxtail millet is highly prized for its nutritional quality, climatic adaptability, and low-input cultivation, and is widely cultivated in many arid and semi-arid regions across Asia, southern Europe, South America, India, and North Africa [27,28]. Furthermore, it displays strong tolerance to drought and saline–alkali stress [29]. The relatively short life cycle and small diploid genome (~500 Mb) of *S. italica* and *S. viridis* render them ideal model systems for cereal and bioenergy crops [30,31]. Switchgrass (*Panicum virgatum*), a perennial warm-season C4 grass native to the North American tallgrass prairies [32], is considered a promising bioenergy crop due to its high productivity, substantial genetic diversity, and broad native range [33]. Furthermore, switchgrass shows strong tolerance to salt, drought, and nutrient deficiency [34]. Sorghum (*Sorghum bicolor*), the fifth most importan**t** cereal crop worldwide and a C_4_ plant, is characterized by high photosynthetic efficiency and an extensive root system. It is an important multipurpose crop in arid and semi-arid regions [35]. Furthermore, *S. bicolor* exhibits strong tolerance to saline–alkali stress [36]. Sorghum [37] and switchgrass [38] have a relatively close phylogenetic relationship to foxtail millet. However, the evolution of FLA genes in four C4 Poaceae plants (foxtail millet, green foxtail, switchgrass and sorghum) and the stress response function of FLA genes in foxtail millet, remain poorly understood. Therefore, the comparison among these four closely related species was intended to clarify *FLA* family composition, orthologous relationships and gene-duplication patterns within the panicoid lineage, thereby providing a framework for the identification of *FLA* genes in foxtail millet.

In this study, we conducted a comprehensive bioinformatics analysis of the FLA gene family in the four species mentioned above. The identified FLA members were systematically examined for gene structure, conserved domains, motifs, chromosomal distribution, phylogenetic relationships, *cis*-acting elements, functional enrichment, and expression patterns under abiotic stresses. Furthermore, the role of *SiFLA6* in saline–alkali tolerance was explored. Through these analyses in a comparative evolutionary context, this study provides valuable insights into the identification of candidate FLA genes associated with growth, development, and abiotic stress responses in these four species.

## 2. Results

### 2.1. Physicochemical Properties of FLA Family Proteins

The *Arabidopsis* FLA protein sequences were used as queries for BLASTp searches against the protein sequences of foxtail millet, green foxtail, sorghum, and switchgrass, respectively. Duplicate sequences were removed, and the HMM profile (PF02469) was downloaded and used with TBtools to screen candidate genes. The intersection of the HMM and BLAST results was retained. Candidate sequences were further validated for their conserved domains using SMART and InterPro. Sequences lacking intact conserved domains or with redundancy were excluded. Consequently, the numbers of *FLA* family members in the four species were determined to be 24, 25, 26, and 46, respectively. Based on their chromosomal positions, these genes were systematically renamed as *SiFLA1-24*, *SvFLA1-25*, *SbFLA1-26*, and *PvFLA1-46*.

Analysis of the physicochemical properties of this gene family (Supplementary Table S1) revealed that amino acid lengths were 192–495 aa, molecular weights (MW) were 19,778.95–51,802.15 Da, and isoelectric points (pI) were 5.02–11.72. Notably, 69 genes (57%) exhibited a pI > 7, indicating an abundance of basic amino acids and designating them as basic proteins. The instability index (II) was 22.09–69.41, with the majority of members scoring > 40, suggesting general structural instability. The aliphatic index (AI) was 70.38–104.38, with higher values implying greater thermostability. Hydropathy prediction based on the grand average of hydropathy (GRAVY) showed that 79 members had GRAVY > 0, classifying them as hydrophobic proteins. Additionally, signal peptide and transmembrane domain prediction revealed that 111 genes harbored signal peptides and 55 genes contained transmembrane regions, implying that this gene family not only facilitates transmembrane transport but may also function as membrane proteins involved in intracellular and extracellular signaling.

### 2.2. Phylogenetic Relationships, Conserved Motifs, and Gene Structure Analysis of the FLA Family

**Figure 1:**
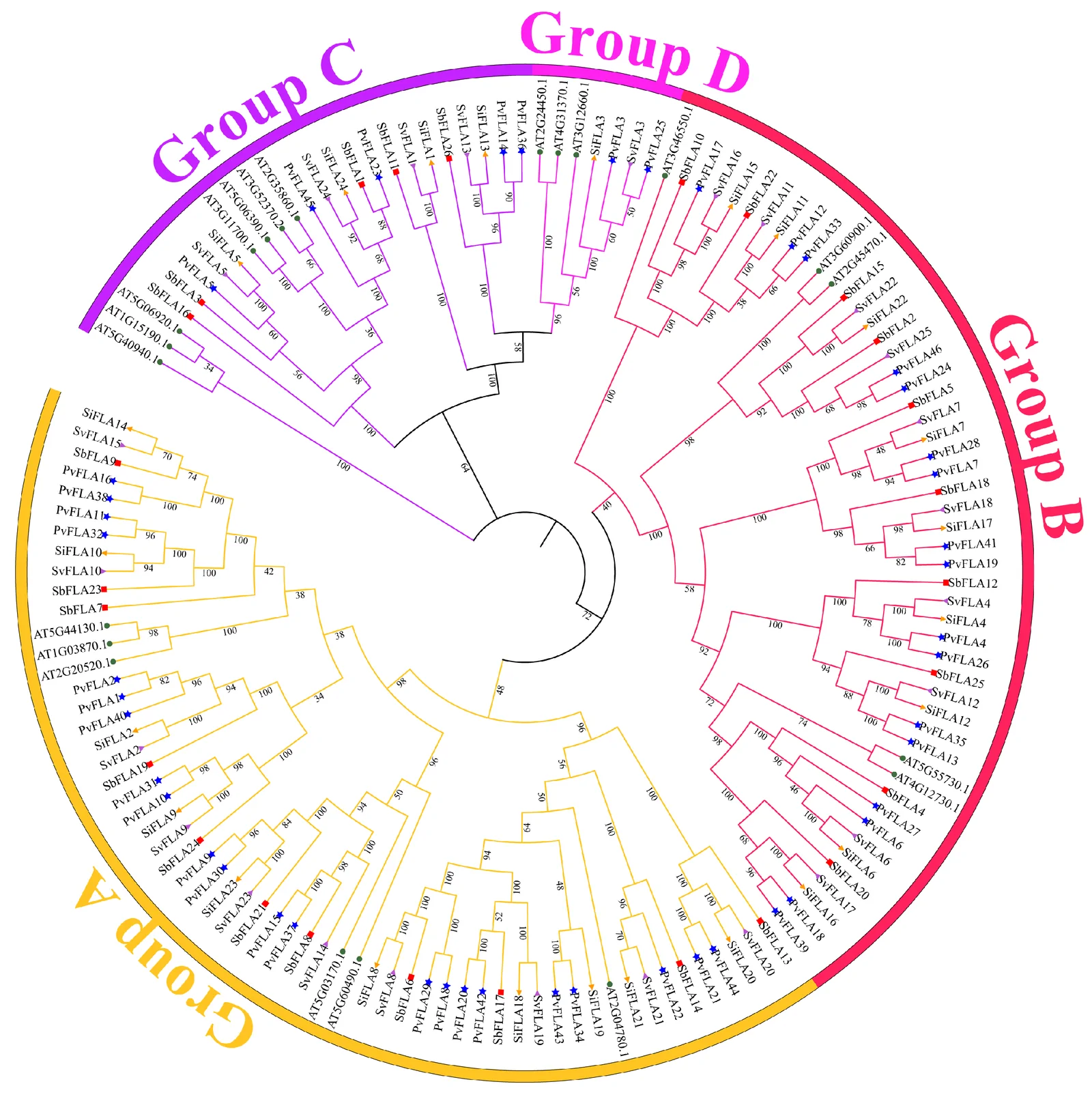
Phylogenetic tree of the *FLA* gene family in foxtail millet, green foxtail, switchgrass, sorghum, and *Arabidopsis*. The tree was constructed using the neighbor-joining (NJ) algorithm in MEGA 11, with bootstrap analysis set to 1000 replicates to assess branch reliability. Different colors represent different groups. A total of 24 *SiFLAs* (left triangles, foxtail millet), 25 *SvFLAs* (right triangles, green foxtail), *46 PvFLAs* (five-pointed stars, switchgrass), *26 SbFLAs* (squares, sorghum), and *21 AtFLAs* (circles, *Arabidopsis*).

A phylogenetic tree was constructed based on 24 *SiFLAs*, 25 *SvFLAs*, 46 *PvFLAs*, 26 *SbFLAs*, and 21 *AtFLAs* (Figure 1). Based on the topology of the phylogenetic tree, all FLA proteins were classified into four subfamilies (Groups A–D), consistent with the classification reported in a previous study [39]. Among them, Group A contained the largest number of members, whereas Group D had the fewest.

To further investigate the functions of the *FLA* gene family, conserved motif and gene structure analysis were performed on all *FLA* genes from the four species (Supplementary Figure S1). In total, ten conserved motifs were identified. In Group A, all members contained motifs 1, 2, 3, 4, and 7, with the exception *SiFLA10*, which lacked motif 3. Additionally, some motifs exhibited subfamily-specific distribution: motif 8 and motif 10 were exclusively present in Group B, whereas motif 5 was restricted to Groups B and C. Furthermore, analysis of gene structures revealed that 42 *FLA* members contained introns. Among them, eight genes possessed introns but lacked untranslated regions (UTRs), whereas another eight lacked both introns and UTRs—notably, the latter group was exclusively found in foxtail millet and green foxtail. Additionally, genes from different species within the same subfamily exhibited similar gene structures, whereas those from the same species but belonging to different subfamilies showed considerable structural variation. This pattern suggests a close evolutionary relationship among members within the same subfamily and likely reflects the relative evolutionary conservation across these four species.

### 2.3. Chromosomal Distribution, Duplication Patterns, and Synteny Analysis of the FLA Family

**Figure 2:**
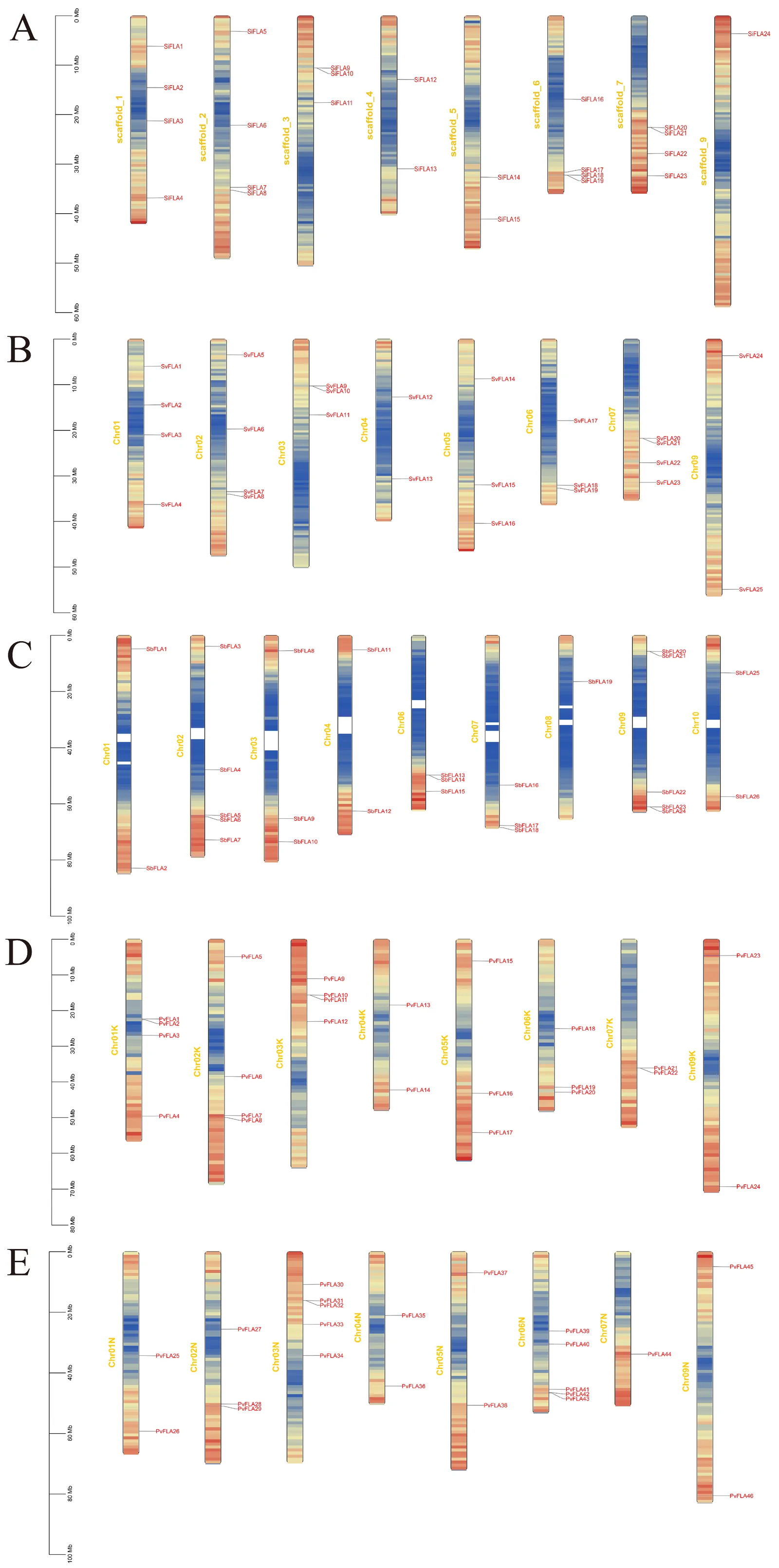
Chromosomal localization of the *FLA* gene family in foxtail millet, green foxtail, switchgrass, and sorghum. (**A**) Chromosomal localization of *SiFLAs*. (**B**) Chromosomal localization of *SvFLAs*. (**C**) Chromosomal localization of *SbFLAs*. (**D**,**E**) Chromosomal localization of *PvFLAs*. Tandem duplication gene clusters, defined as adjacent *FLA* genes located on the same chromosome, are marked by leader lines. The varying colors of chromosomes indicate gene density.

To elucidate the chromosomal distribution of *FLA* genes in foxtail millet, green foxtail, switchgrass, and sorghum, their genomic locations were mapped (Figure 2). Both *SiFLAs* and *SvFLAs* were distributed across nine chromosomes. In both species, chromosome 9 harbored the fewest genes, and no *FLA* genes were detected on chromosome 8 (Figure 2A, B). The *SbFLAs* were distributed on ten chromosomes, with no genes on chromosome 5, and the most genes were clustered at the proximal and distal ends, leaving the central regions devoid of *FLA* genes (Figure 2C). In switchgrass, the *PvFLAs* were unevenly distributed across 18 chromosomes, with no genes found on chromosomes Chr08K and Chr08N (Figure 2D). Furthermore, tandem gene clusters were identified in all four species. Notably, the gene clusters in both *SiFLAs* and *SvFLAs* were located on chromosomes 3, 6, and 7: for instance, *SiFLA9* and *SiFLA10* were clustered on chromosome 3 of foxtail millet, and *SvFLA9* and *SvFLA10* formed a tandem cluster on chromosome 3 of green foxtail, suggesting a conserved chromosomal distribution pattern between foxtail millet and green foxtail.

To investigate the expansion patterns of the *FLA* gene family, duplication events of *FLA* genes in foxtail millet, green foxtail, switchgrass, and sorghum were analyzed (Supplementary Figure S2). A total of 75 duplicated gene pairs were identified, comprising 63 segmental and 12 tandem duplication pairs. To further elucidate the evolutionary process of the *FLA* gene family, the Ka/Ks ratios of these duplicated gene pairs were calculated (Supplementary Table S2). The majority of duplicated *FLA* gene pairs exhibited Ka/Ks values of less than 1, suggesting that the *FLA* family has been under strong purifying selection during evolution.

**Figure 3:**
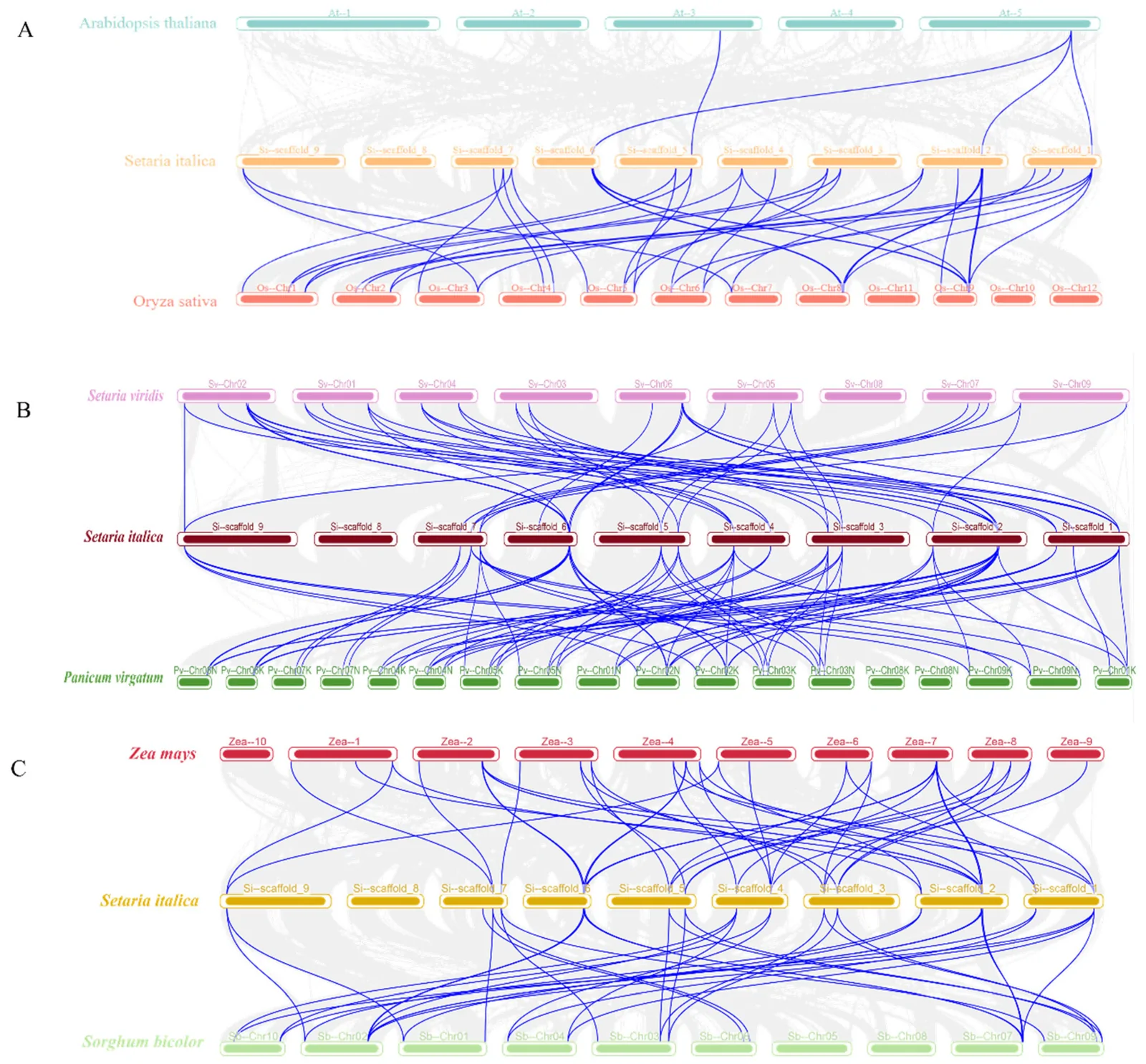
Collinearity analysis of the *FLA* gene families across multiple species. (**A**) Correlation analysis of foxtail millet with *Arabidopsis thaliana* and rice. (**B**) Correlation analysis of foxtail millet with green foxtail and switchgrass. (**C**) Correlation analysis of foxtail millet with corn and sorghum. The faint grey lines in the background represent all syntenic blocks across the genomes, while blue lines denote collinear *FLA* gene pairs between different species. Chromosome bars are drawn in accordance with their physical positions in the genome. The collinear *FLA* gene pairs discussed in the main text correspond to these blue connecting lines. From left to right, the genes enclosed in red boxes are *SiFLA17*, *SiFLA15*, *SiFLA7* and *SiFLA4*, respectively.

Synteny analysis was performed across seven species—foxtail millet, green foxtail, switchgrass, sorghum, *Arabidopsis*, rice, and maize—to investigate the phylogenetic relationships of the *FLA* gene family (Figure 3). Switchgrass shared the highest number of homologous gene pairs with foxtail millet (70 pairs), whereas *Arabidopsis* had the fewest (only 4 pairs). Notably, no homologous gene pairs were detected between chromosome 8 of foxtail millet and the other six species. Furthermore, *SiFLA4*, *SiFLA7*, *SiFLA15*, and *SiFLA17* possessed homologs in all six other species. (Supplementary Table S3). Additionally, some of these genes participated in at least two homologous gene pairs, suggesting that they may have played important roles during plant evolution.

### 2.4. Evolutionary Relationships and Cis-Acting Element Analysis of the FLA Family

**Figure 4:**
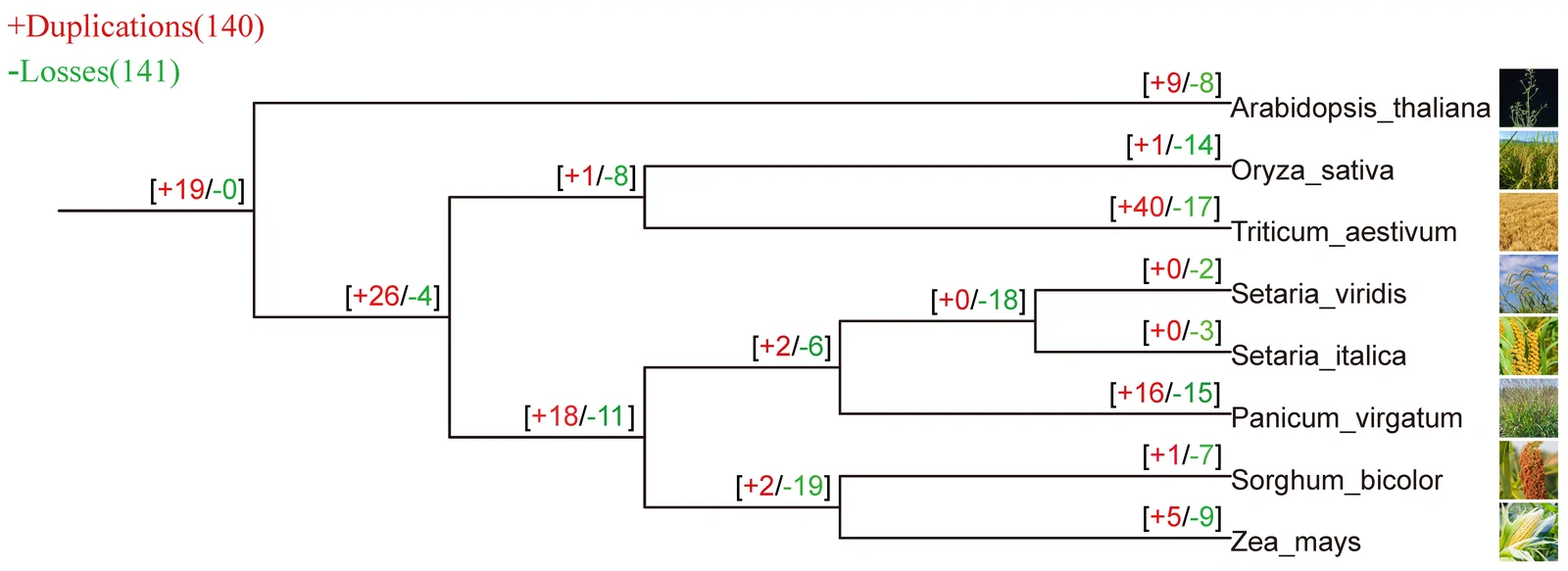
Analysis of the gain and loss of family members during the evolution of the *FLA* gene family in foxtail millet, green foxtail, switchgrass, sorghum, *Arabidopsis*, rice, wheat and corn.

To investigate the gene gain and loss events of the *FLA* gene family during evolution, the evolutionary dynamics were analyzed based on eight species (foxtail millet, green foxtail, switchgrass, sorghum, *Arabidopsis*, rice, maize, and wheat) (Figure 4). The total number of lost *FLA* genes across these species was 141, which was slightly greater than the total number of gained genes (140). This suggests that the *FLA* gene family has undergone a gradual contraction over evolutionary time. At the taxonomic level, the *FLA* gene family expanded by 19 genes in dicots with no losses, whereas in monocots, 26 genes were gained and 4 were lost. Overall, this indicates that gene loss is more pronounced in monocots during the differentiation of the *FLA* gene family. At the species level, the number of *FLA* genes increased during evolution in *Arabidopsis*, wheat, and switchgrass. Among these three species, wheat exhibited the most substantial expansion, with a net gain of 40 genes and a loss of 17. By contrast, the other five species experienced gene loss during evolution, with rice showing the most severe contraction—gaining only 1 gene while losing 14.

To investigate the regulatory mechanisms of the *FLA* gene family, *cis*-acting elements in the promoter regions (2000 bp upstream of the transcription start site) of the *FLA* genes were predicted (Supplementary Figure S3). According to their functions, the *cis*-acting elements identified in all *FLA* members were categorized into four classes. Class I contained light-responsive elements. Class II contained hormone-responsive elements, such as auxin and gibberellin-responsive elements. Class III contained stress-responsive elements, including low-temperature-responsive elements and MYB binding sites involved in drought induction. Class IV contained *cis*-acting elements associated with growth and development, such as those associated with meristem or endosperm expression. Notably, most *FLA* members contained multiple identical *cis*-acting elements, suggesting that these genes are regulated by various factors and potentially through synergistic or antagonistic interactions among different members. Furthermore, abscisic acid (ABA)-responsive, low-temperature-induced, and meristem expression-related elements were present in almost all *FLA* genes. This indicates that *FLA* genes not only respond to abiotic and hormonal stresses but also play crucial roles in plant growth and development.

### 2.5. Expression Patterns of SiFLAs Under Diverse Abiotic Stresses and GO Enrichment Analysis

**Figure 5:**
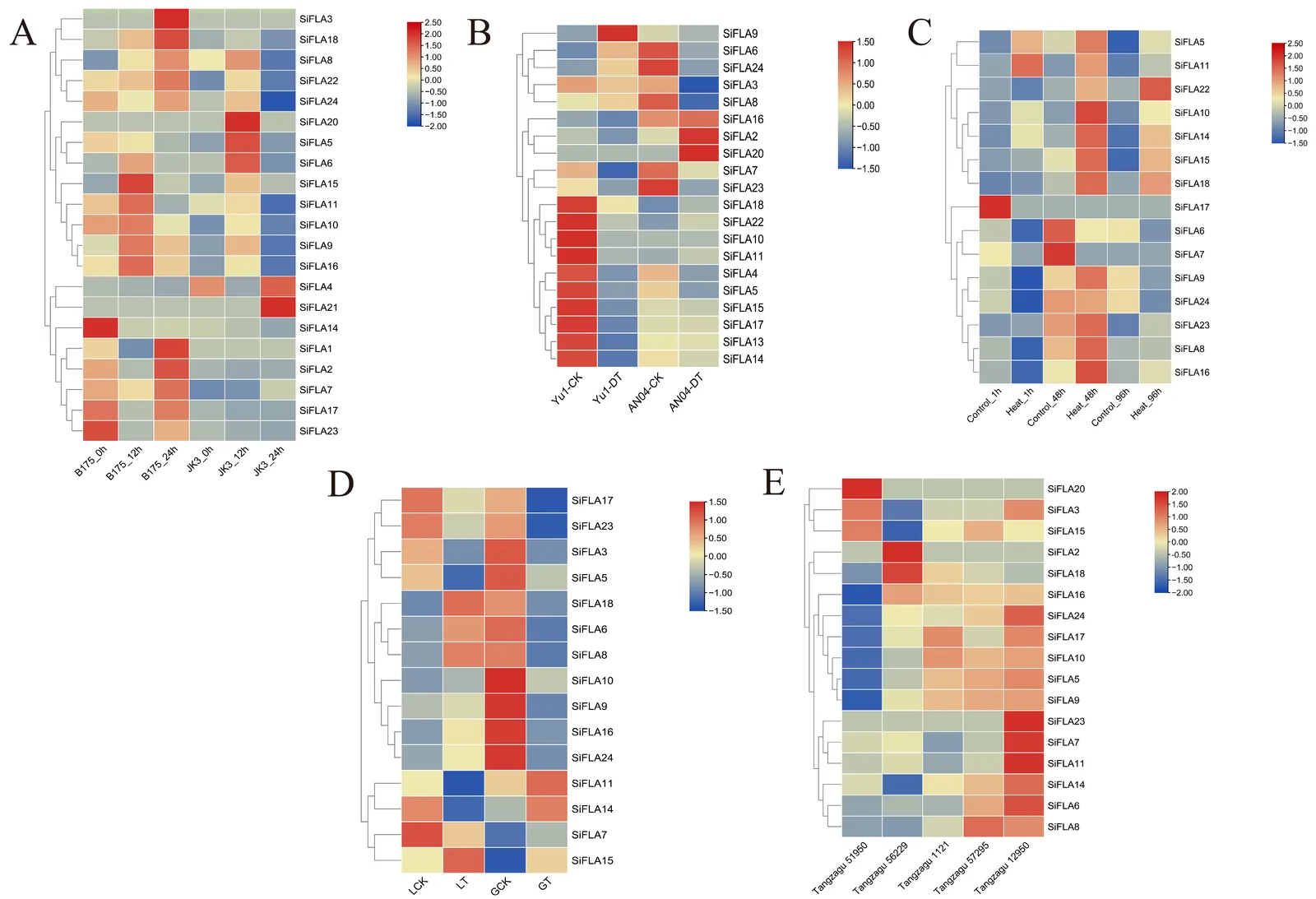
Expression analysis of *SiFLAs* under five abiotic stresses. (**A**) Expression analysis under saline–alkali stress. (**B**) Expression analysis under drought stress. Yu1-CK and AN04-CK represent the drought-tolerant cultivar ‘Yugu1’ and the drought-sensitive cultivar AN04 under the control treatment, respectively; Yu1-DT and AN04-DT represent ‘Yugu1’ and AN04 under the drought-stress treatment, respectively. (**C**) Expression analysis under high-temperature stress. Control and heat represent the control and heat-stress treatments, respectively. (**D**) Expression analysis under herbicide stress. GCK and LCK represent the atrazine-resistant cultivar GA2 and the atrazine-sensitive cultivar LG31 under the control treatment, respectively; GT and LT represent GA2 and LG31 under atrazine stress, respectively. (**E**) Expression analysis under shading stress. Two shade-tolerant cultivars (Tangzagu56229 and 51950) and three shade-sensitive cultivars (Tangzagu12950, 1121 and 57295) under shade stress. The color scale of the heatmap shows gene expression after row-wise Z-score normalization. The input data were averaged FPKM values from biological replicates. Red indicates relatively high expression, while blue indicates relatively low expression of each gene across different stress treatments.

To validate the functions of *SiFLAs* under saline–alkali stress, the expression levels (FPKM) in a saline–alkali sensitive foxtail millet cultivar, B175, and a saline–alkali tolerant cultivar, JK3, were analyzed at 0, 12, and 24 h under treatment. A total of 21 expressed genes were detected (Figure 5A). The expression levels of *SiFLA5*, *SiFLA6*, and *SiFLA20* were higher in JK3 at 12 h, whereas *SiFLA4* and *SiFLA21* exhibited high expression at 24 h. Meanwhile, these genes were consistently low in B175. These genes were thus selected as candidates for subsequent functional validation.

To investigate the response mechanisms of *SiFLAs* under drought, high-temperature, shade, and herbicide-induced stresses, transcriptomic datasets under these four abiotic stress conditions were retrieved and subjected to expression analysis. A total of 20 expressed *SiFLAs* were detected under drought stress (Figure 5B). In the drought-tolerant foxtail millet cultivar Yugu 1, *SiFLA9* showed higher expression levels after drought treatment than before treatment. Under high-temperature stress, a total of 15 *SiFLAs* were detected (Figure 5C). *SiFLA11* showed higher expression at 1 h, whereas *SiFLA22* showed higher expression at 96 h. Under herbicide stress, 15 *SiFLAs* were identified (Figure 5D). After treatment, *SiFLA11* and *SiFLA14* showed higher expression levels in the atrazine resistant cultivar Gongai2 (GA2). Under shade stress, a total of 17 *SiFLAs* were detected (Figure 5E). After shade exposure, *SiFLA20*, *SiFLA3*, *SiFLA15*, *SiFLA2*, and *SiFLA18* showed higher expression levels in the shade-tolerant cultivars Tangzagu 56229 and Tangzagu 51950.

**Figure 6:**
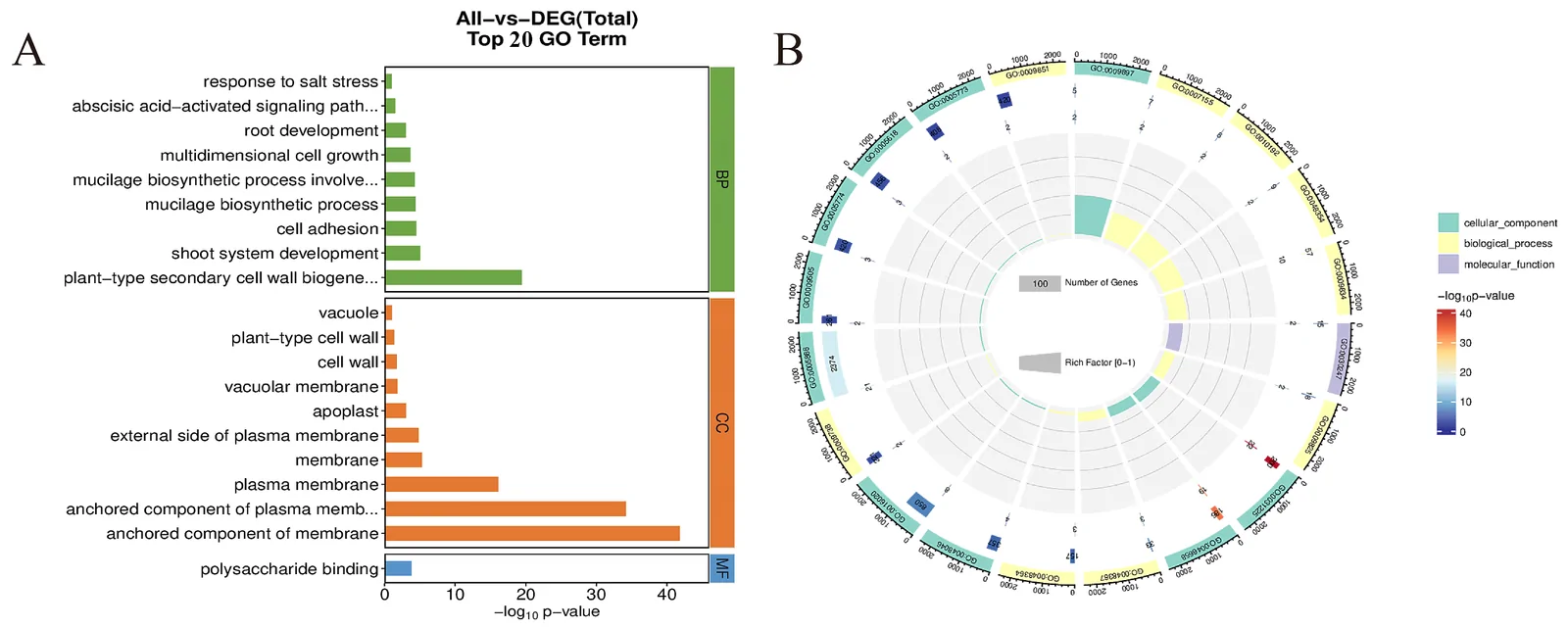
GO enrichment analysis of *SiFLAs*. (**A**) Histogram showing the top 20 significantly enriched GO terms of SiFLA genes. The *x*-axis represents −log10 (*p*-value), reflecting the significance of enrichment, and the *y*-axis shows GO functional terms. The bar color corresponds to three main GO categories: biological process (BP), cellular component (CC), and molecular function (MF). (**B**) Circular plot illustrating all enriched GO terms. The size of inner squares indicates the number of enriched genes, and the color gradient represents the rich factor ranging from 0 to 1. GO terms are grouped into BP, CC and MF categories as shown in the outer ring.

GO enrichment analysis was performed on *SiFLAs* (Figure 6). These genes were enriched in 20 GO terms across three main categories: biological process (BP), cellular component (CC), and molecular function (MF). For BP, SiFLAs were primarily enriched in plant-type secondary cell wall biogenesis and root development; for CC, the enriched terms were mainly associated with membrane anchoring and the plasma membrane; and for MF, the genes were predominantly enriched in polysaccharide binding. Based on their *p*-values, the top 20 enriched GO terms were analyzed. The highest number of genes was associated with the membrane-anchoring component, with a *p* < 0.05.

### 2.6. Cloning, Phylogenetic Analysis, and Subcellular Localization of SiFLA6

Expression profiles under saline–alkali stress showed that *SiFLA4/5/6/20/21* expressed at relatively high levels. Therefore, using JK3 cDNA as the template, the five genes were cloned, but only the CDS sequence of *SiFLA6* could be obtained (Supplementary Figure S4A), whereas the other four genes could not be amplified. Moreover, *SiFLA6* showed relatively high expression in both B175 and JK3 at 12 h after stress, with the higher expression in JK3, suggesting that it may be an important gene in response to saline–alkali stress. Therefore, *SiFLA6* was selected for the subsequent functional analyses. Then, a 1302-2Myc-eGFP-*SiFLA6* recombinant fusion expression vector was constructed (Supplementary Figure S4B), and both the gene sequence and the recombinant vector were confirmed by sequencing.

**Figure 7:**
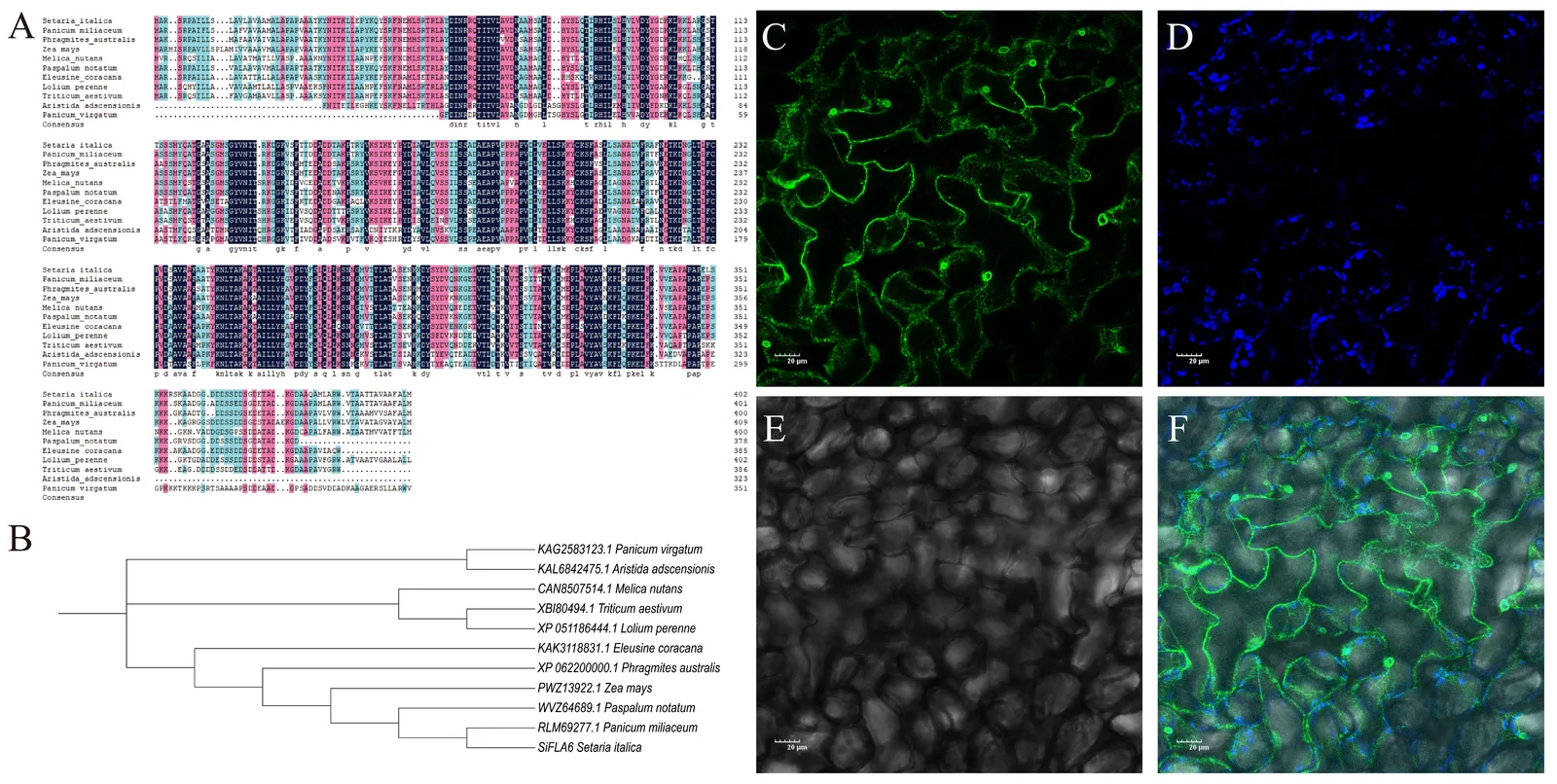
Sequence homology alignment and subcellular localization of SiFLA6. (**A**) Multiple sequence alignment of FLA homologous proteins from different plants. Different colors represent different amino acid residues, and the black background indicates the highly conserved sequence regions. (**B**) Phylogenetic tree of FLA homologous proteins from different plants. (**C**–**F**) Confocal laser scanning microscopy images for subcellular localization assay. (**C**) eGFP fluorescence channel; (**D**) Chlorophyll autofluorescence channel; (**E**) Bright-field channel; (**F**) Merged image of eGFP, chlorophyll autofluorescence and bright-field signals.

To characterize the sequence features of *SiFLA6* and its evolutionary relationships with homologous genes in other species, the protein sequences of *SiFLA6* and its homologs were aligned, and a phylogenetic tree was constructed (Figure 7A,B). *SiFLA6* shares conserved domains with homologous proteins in *Panicum miliaceum*, *Phragmites australis*, *Zea mays*, *Melica nutans*, *Paspalum notatum*, *Eleusine coracana*, *Lolium perenne*, *Triticum aestivum*, *Aristida adscensionis*, and *Panicum virgatum*. Sequence alignment revealed substantial variations in the N- and C-terminal regions, with relatively low homology and marked sequence variation across species. In contrast, the central region was highly conserved. Phylogenetic analysis showed that *SiFLA6* has the closest evolutionary relationship with the protein RLM69277.1 in *Panicum miliaceum*.

To determine the subcellular localization of the SiFLA6 protein in plant cells, subcellular localization analysis was performed using the OE-1 line (Figure 7C–F). SiFLA6 was localized to the endoplasmic reticulum.

### 2.7. Functional Analysis of SiFLA6 in Saline–Alkali Tolerance

**Figure 8:**
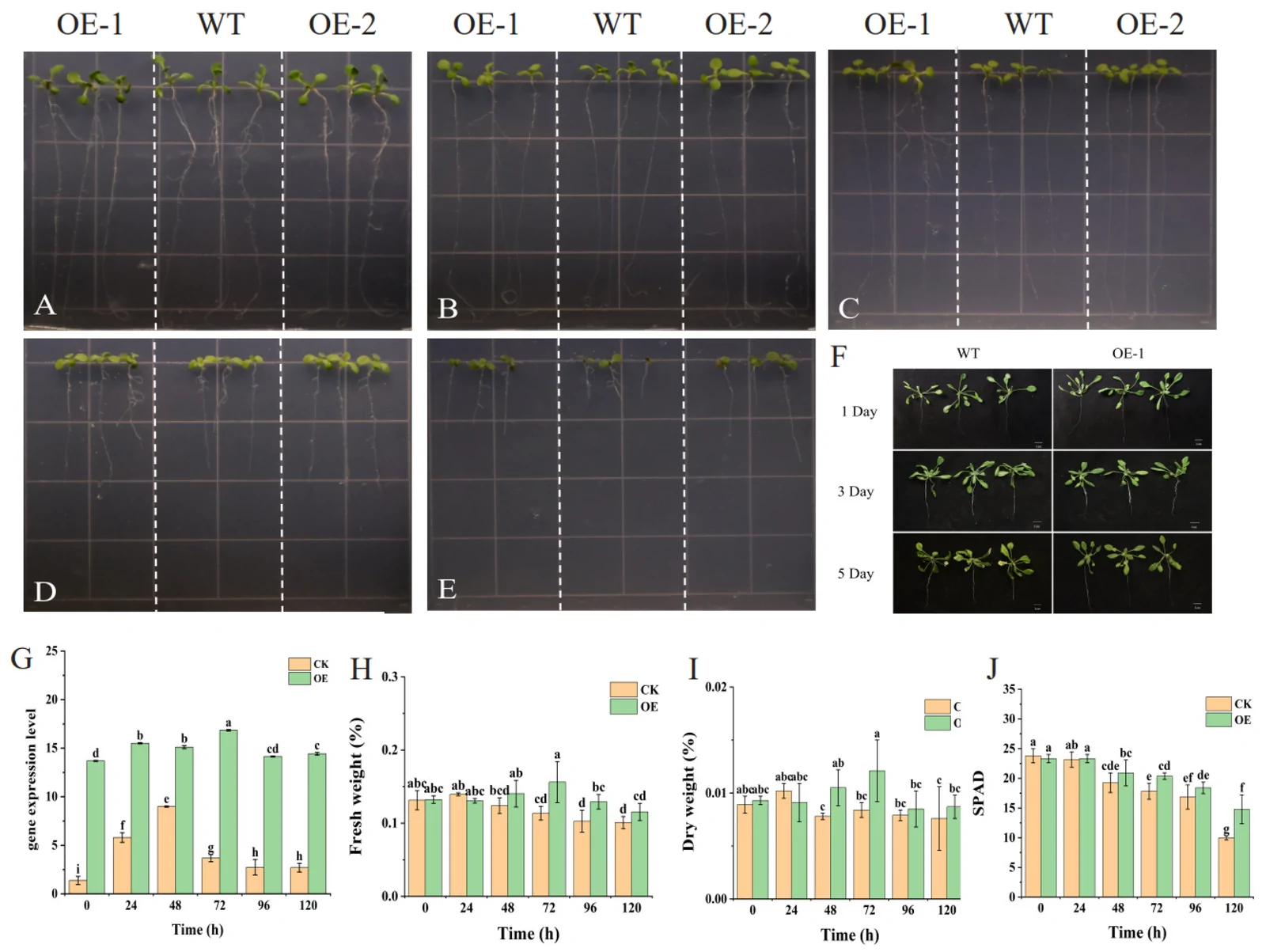
Root vigor, morphology, and gene expression of WT and OE lines under saline–alkali stress. (**A**–**E**) shows the effects of 0%, 10%, 20%, 30% and 40% seawater stress treatments on seedling-stage OE-1, WT and OE-2, respectively. Every three seedlings from left to right represent one line, namely OE-1, WT and OE-2. (**F**) Morphological changes in WT and OE-1 on the 1st, 3rd and 5th days after 50% seawater stress treatment. (**G**) qRT-PCR detection of *SiFLA6* gene expression levels. CK stands for WT, and OE stands for OE-1; the same applies hereinafter. (**H**–**J**) Fresh weight, dry weight, and SPAD values of WT and OE-1 at different time points under saline–alkali stress. The data are presented as mean ± standard deviation (SD). Statistical analyses were performed using one-way analysis of variance (ANOVA) followed by the LSD test. *p* < 0.05 was set as the threshold for significant difference. Three independent biological replicates were performed (*n* = 3). Different letters above bars indicate significant differences, whereas bars sharing the same letter are not significantly different.

To investigate the response of *SiFLA6* to saline–alkali stress, the root growth of the OE-1, OE-2, and wild-type (WT) lines at the germination stage was analyzed under different seawater concentration (0%, 10%, 20%, 30%, and 40%). Under control conditions (0% seawater), there was no apparent difference in root length between the WT and OE lines. As the seawater concentration increased, root length inhibition was more pronounced in the WT line. In contrast, the root lengths of OE-1 and OE-2 were greater than those of the WT across all seawater concentrations, with the most pronounced differences observed at 40% seawater (Figure 8A–E). These results suggest that overexpression of *SiFLA6* enhances the tolerance of *Arabidopsis* to seawater stress.

The root systems of OE-1 and OE-2 both exhibited stronger tolerance compared with the WT. Because of the limited number of OE-2 seeds, we chose OE-1 for further stress experiments at the seedling stage to investigate the function of *SiFLA6* under saline–alkali stress (50% seawater). The expression levels of *SiFLA6* in OE-1 line were analyzed at 0, 24, 48, 72, 96, and 120 h under 50% seawater stress (Figure 8G). In the OE-1 line, *SiFLA6* exhibited an extremely high basal expression level at 0 h, and maintained high expression throughout the stress period. These findings confirm that *SiFLA6* is efficiently expressed in the OE-1 line. To further investigate the effects of *SiFLA6* on the growth of *Arabidopsis* seedlings, the fresh weight, dry weight, and leaf SPAD values were analyzed under 50% seawater stress (Figure 8H–J). As stress duration increased, the WT plants showed leaf yellowing and wilting. Although the OE-1 line also showed some stress damage, the symptoms were substantially milder than those in the WT; the leaves remained relatively green, and the plant morphology was better maintained (Figure 8F). After 72 h of stress treatment, both the fresh and dry weights of the WT and OE-1 began to decrease, but the values for OE-1 were significantly higher than those of the WT. Although the SPAD values of both WT and OE-1 showed a decreasing trend, the decline in OE-1 was much less severe than that in the WT. These results indicate that overexpression of *SiFLA6* can significantly alleviate the inhibitory effect of seawater stress on *Arabidopsis* growth, thereby possibly maintaining better photosynthetic capacity.

### 2.8. Physiological Mechanisms Underlying SiFLA6-Mediated Saline–Alkali Tolerance

**Figure 9:**
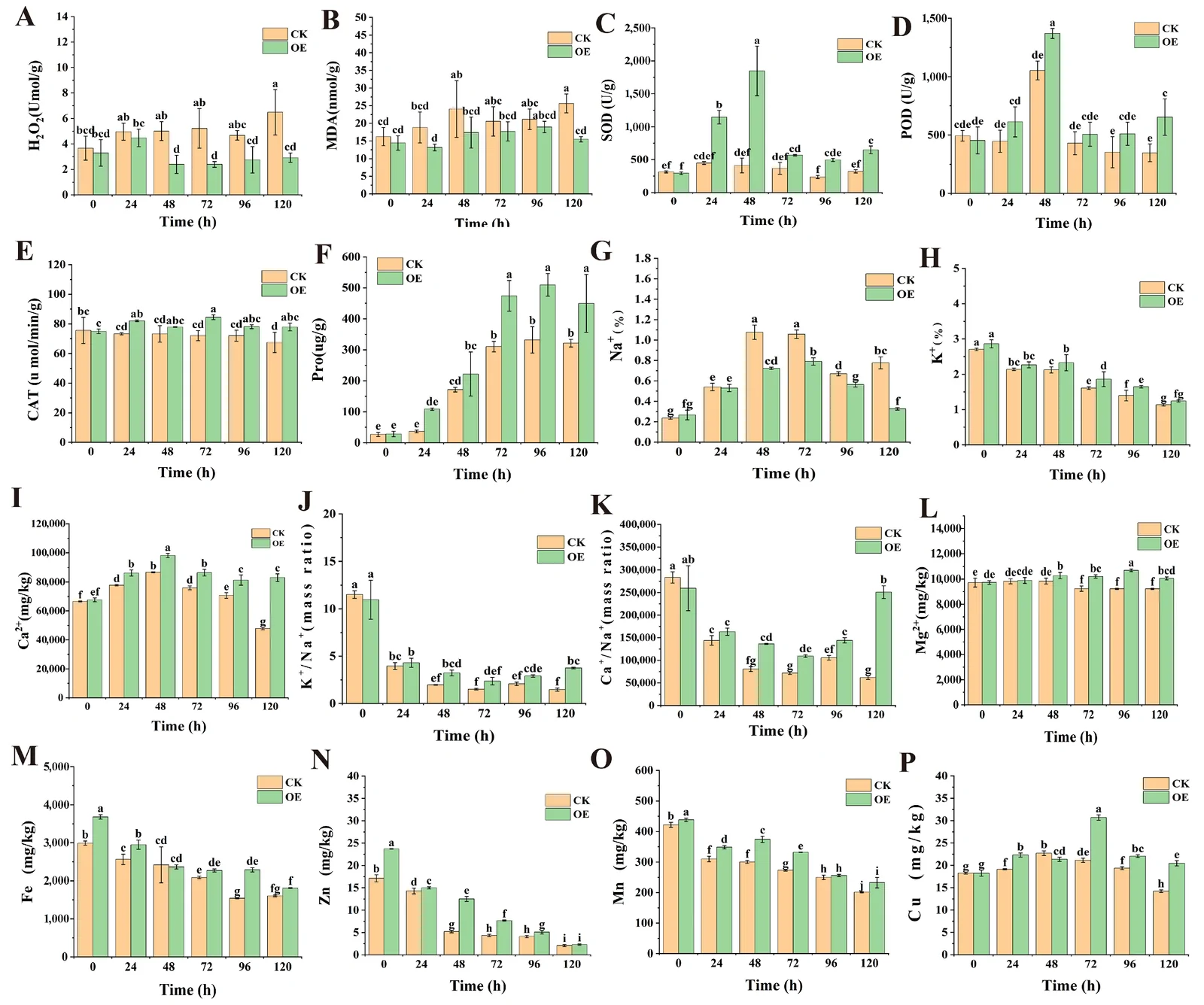
Physiological and ionomic responses of WT and OE-1 to saline–alkali stress. (**A**–**F**) Contents of H_2_O_2_, MDA, SOD, POD, CAT and Pro in WT and OE-1 under saline–alkali stress. (**G**–**P**) Contents of ions and trace elements in WT and OE-1 under saline–alkali stress. The data are shown as mean ± standard deviation (SD). One-way ANOVA followed by the LSD test was used for statistical analysis, with a significance threshold of *p* < 0.05. Three independent biological replicates were performed (*n* = 3). Different letters above bars indicate significant differences, whereas bars sharing the same letter are not significantly different.

To investigate the physiological mechanisms underlying the enhanced seawater tolerance conferred by *SiFLA6* in *Arabidopsis*, the regulation of the antioxidant defense system by *SiFLA6* was first analyzed (Figure 9A–F). Under stress conditions, the H_2_O_2_ and MDA levels in both WT and OE-1 increased. However, the increase was significantly lower in OE-1 than those in the WT. At 120 h, the H_2_O_2_ and MDA contents in OE-1 were 45% and 61% lower than those of the WT, respectively, suggesting reduced membrane lipid peroxidation and oxidative damage in the OE-1 line. Further analysis showed that under stress, the activities of SOD, POD, and CAT in both WT and OE-1 initially increased and then decreased. However, those in OE-1 were significantly higher than those in the WT starting at 24 h. By 120 h, the SOD and POD activities in OE-1 remained 2-fold those of the WT, and CAT activity remained 1.2-fold, indicating that OE-1 possesses a stronger and more sustained capacity for scavenging reactive oxygen species and thus experiences reduced oxidative damage. Proline (Pro) content increased gradually under stress. The Pro content in OE-1 was significantly higher than that in the WT throughout the stress period, reaching a peak at 96 h, which was 1.6-fold higher than that of the WT. This indicates that OE-1 has a stronger osmotic adjustment ability.

Additionally, under seawater stress, the Na^+^ level in both WT and OE-1 increased and then decreased. The accumulation of Na^+^ in OE-1 was significantly lower than that in the WT, and by 120 h, the Na^+^ content in OE-1 was 42% lower than that in the WT. In contrast, the K^+^ level decreased in WT, whereas OE-1 maintained a higher K^+^ level. Consequently, the K^+^/Na^+^ ratio in OE-1 remained higher than that in the WT throughout the stress period, reaching 2.5-fold that of WT at 120 h. The Ca^2+^ level in both WT and OE-1 increased and then decreased. However, during the late stage of stress, the Ca^2+^ level in OE-1 remained stable, whereas it decreased significantly in WT, leading to a significantly higher Ca^2+^/Na^+^ ratio in OE-1 than in WT (Figure 9G–K). During the mid-to-late stress period, the levels of Mg^2+^, Fe, Zn, and Mn declined significantly in both WT and OE-1. However, OE-1 maintained these trace elements at relatively high levels, which were consistently higher than those in WT. Cu content was maintained at higher levels in OE-1, with its content during the late stress period significantly higher than at 0 h or than that in the WT. This may be closely associated with the roles of Cu in ABA synthesis and Cu/Zn-SOD activity (Figure 9L–P).

## 3. Discussion

### 3.1. Evolutionary Characteristics of the FLA Gene Family in Four Poaceae Species

FLAs belong to a subclass of hydroxyproline-rich glycoproteins (HRGPs). Increasing evidence indicates that they play crucial roles in secondary cell wall formation and stress responses [40]. However, most studies have predominantly focused on their functions in herbaceous plants, particularly *Arabidopsis*, whereas their functions in Poaceae species remain largely unexplored. A point of debate regarding the physicochemical properties of FLAs concerns the presence of an N-terminal signal peptide. All FLA proteins in some plant species, such as *Arabidopsis*, cabbage, and cotton, contain signal peptides [8,41,42]. However, specific members in wheat, rice, and eucalyptus lack this sequence [20,43]. In this study, the majority of FLA family members across the four investigated species contained signal peptides, suggesting that these members may share high homology with those of *Arabidopsis*. Although the number of *FLA* members in each of our four investigated species was greater than that of *Arabidopsis*, the number of group C members in all four species was lower than in *Arabidopsis*. This reflects the rich diversity of the *FLA* gene family in higher plants, which is consistent with the findings of Zang et al. [10]. In this study, the gene structures of *FLA* genes in the four species were analyzed. The majority of members had lost their introns, with only 33% containing introns. In *Salicaceae* (*Populus and Salix*), 34.88% of FLA genes contain introns, and a similar proportion was reported in tobacco [39,40], suggesting that introns in FLA genes are generally lost during plant evolution.

Segmental and tandem duplications are the primary driving forces for gene family expansion [44]. The larger FLA family in switchgrass is most parsimoniously explained by its allotetraploid origin (K and N subgenomes; Figure 2D,E), rather than by an accelerated evolutionary rate [45,46]. The Ka/Ks ratios of most duplicated FLA pairs were below 1 (Supplementary Table S2), indicating purifying selection and functional conservation after duplication, whereas a few pairs with Ka/Ks slightly above 1 may reflect relaxed selection or weak positive selection. FLA gene families have been widely characterized across terrestrial plants, and phylogenetic analysis, conserved motif prediction, and related analyses are routine for such studies. The FLA family has expanded mainly through gene duplication, and orthologous FLA genes retained across species are mostly under purifying selection with potential conserved functions [7]. Our evolutionary analysis identified *SiFLA4*, *SiFLA7*, *SiFLA15*, and *SiFLA17* as cross-species conserved genes. The biological functions of these four conserved genes remain to be further investigated in future work.

### 3.2. SiFLAs Are Involved in Abiotic Stress Responses

In this study, analysis of promoter *cis*-acting elements revealed that the promoters of *FLA* genes in the four species contain various *cis*-acting elements associated with hormone responses, stress responses, light responses, and plant growth and development. This suggests that *FLA* genes may possess broad biological functions and are involved in diverse metabolic processes [47]. Moreover, biotic and abiotic stresses induce significant transcriptional changes in *FLA* genes, enabling active responses to stress conditions. For instance, *AtFLA9* was downregulated under drought conditions [48]; exogenous abscisic acid (ABA) treatment strongly reduced the expression of *OsFLA24* and *AtFLA1/2/8* [3,8]; and *SlFLA15* and *SlFLA17* were downregulated upon ABA treatment, suggesting that they are involved in the ABA signaling pathway. Under H_2_O_2_ stress, the expression levels of wheat FLA genes were upregulated, which may contribute to the tolerance of wheat to H_2_O_2_ [49]. In other species, *FLA* genes respond to various abiotic stresses. For example, the expression levels of *SlFLA1* and *SlFLA14* in tomato were significantly increased under dark, low-temperature, and salt stresses [50]. In this study, the response mechanisms of *SiFLA* genes to five abiotic stresses, including saline–alkali stress, high temperature, drought, shade, and herbicide stress, were investigated. We found that the expression levels of most *SiFLA* genes were affected under specific abiotic stresses. This suggests the functional diversity of *FLA* genes. Furthermore, *SbFLA* genes showed distinct temporal expression patterns under saline–alkali stress [51]. Consistently, our study revealed that some *SiFLA* genes showed temporally specific expression under saline–alkali stress; e.g., some exhibited high expression at 12 h and low expression at 24 h. This may reflect an initial stress response to saline–alkali stress, followed by an adaptive phase. A similar phenomenon has been observed in *ZmFLA* genes [52]. The differential temporal expression patterns of *SiFLA* genes further support the core roles of *FLA* genes in plant abiotic stress responses.

### 3.3. SiFLA6 Enhances Saline–Alkali Tolerance

*FLA* genes play crucial roles in plant salt stress responses. For example, under salt stress, the expression of six *FLA* genes in poplar, e.g., *PtrFLA2*, was upregulated, whereas the expression of rice *FLA* genes *OsFLA10* and *OsFLA18* was downregulated [3,10]. In this study, the OE-1 line maintained high *SiFLA6* expression throughout the saline–alkali stress period, consistent with the stress-responsive nature of FLA genes, suggesting that *FLA* genes in different species may exhibit distinct regulatory patterns in response to salt stress. In this study, upregulation of *SiFLA6* promoted root growth vigor, fresh weight, and dry weight in OE-1. This result is consistent with the observation that overexpression of the *phaC1* gene leads to higher biomass and the *AtFLA4* mutant inhibits root growth under salt stress [53,54]. Photosynthesis, a critical and complex physiological process in plants, is severely impacted by various abiotic stresses. The SPAD value is an important indicator reflecting chlorophyll content and is closely related to photosynthetic capacity [55]. In this study, upregulation of *SiFLA6* increased the SPAD values in OE-1 during the late stage of saline–alkali stress, indicating a superior photosynthetic capacity compared WT. These findings suggest that *SiFLA6* acts as a positive regulator in the saline–alkali stress response. *SiFLA6* may effectively alleviate the inhibitory effect of saline–alkali stress on plant growth by regulating root growth and leaf photosynthetic capacity. This is consistent with previous conclusions that *FLA* family genes are widely involved in plant salt stress responses, thus further enriching the functional research of the *FLA* gene family in saline–alkali stress.

Under stress conditions, reactive oxygen species (ROS) accumulate to excessive levels in plants, leading to oxidative damage. MDA content is regarded as a physiological marker of the lipid peroxidation rate, and H_2_O_2_ is a key ROS molecule [56]. Both are key physiological indicators for assessing oxidative damage in plants [57]. In this study, the H_2_O_2_ and MDA contents in OE-1 were significantly lower than those in the WT, indicating reduced membrane lipid peroxidation. To counteract oxidative damage, the activities of antioxidant enzymes such as SOD, CAT, and POD increased significantly. These enzymes coordinately scavenge excess ROS, alleviating oxidative damage and thus protecting the integrity of cell membrane structure and function [58]. In this study, OE-1 enhanced the activities of these antioxidant enzymes (SOD, POD, and CAT), thereby reducing ROS accumulation and improving the saline–alkali tolerance of *Arabidopsis*. This is consistent with the observation that overexpression of *CeqHAK6* and *CeqHAK11* in *Casuarina equisetifolia* significantly enhanced the salt tolerance of *Arabidopsis* [59]. Furthermore, Pro is a key osmolyte for maintaining osmotic balance and improving saline–alkali tolerance in plants [60]. In this study, OE-1 accumulated higher Pro levels, enhancing osmotic adjustment. Similar results were reported in *Arabidopsis* overexpressing the soybean gene *GmEF4*, which exhibited reduced H_2_O_2_ and MDA levels and increased Pro content after stress treatment [61]. Additionally, overexpression of *IbHAK11*, *MeHKT1*, and *AtKUP9* in *Arabidopsis* increased soluble sugars, Pro, and other osmolyte contents [62,63,64]. Notably, the antioxidant advantage of OE-1 persisted during the late stress stage (96–120 h), indicating that OE-1 confers sustained rather than transient antioxidant capacity. This is consistent with previous reports on salt tolerance genes such as *OsSOS1* [65].

Plants have evolved strategies to maintain ion balance under saline–alkali stress by preventing Na^+^ influx and promoting K^+^ uptake, thereby preserving Na^+^ and K^+^ homeostasis in the cytoplasm. A high K^+^/Na^+^ ratio is critical for maintaining normal cellular metabolism and serves as a key indicator for evaluating salt tolerance [66,67]. Previous studies have shown that overexpression of *ZmVP1* from *Zoysia matrella* in *Arabidopsis* resulted in a significantly higher K^+^/Na^+^ ratio in leaves under salt stress than in WT [68]. Overexpression of *AtABCG36* in *Arabidopsis* enhanced both drought and salt tolerance by reducing Na^+^ accumulation [69]. Under salt stress, *SpCIPK2* accelerated Na^+^ efflux and mitigated K^+^ efflux in transgenic *Arabidopsis*, leading to reduced Na^+^ accumulation and an elevated K^+^/Na^+^ ratio [70]. In this study, the Na^+^ accumulation was significantly lower in OE-1 than in WT, whereas the K^+^ content showed a smaller decrease. Consequently, the K^+^/Na^+^ ratio in OE-1 remained significantly higher than in WT. These results suggest that OE-1 improved Na^+^/K^+^ homeostasis and a higher K^+^/Na^+^ ratio, which may contribute to the saline–alkali tolerance. Ca^2+^ signaling functions as a second messenger in response to external stimuli and is a core signaling molecule in plant salt stress responses. It promotes Na^+^ extrusion by activating the *SOS3/CBL4-SOS2/CIPK24-SOS1* signaling pathway [71]. In this study, OE-1 maintained a higher Ca^2+^/Na^+^ ratio. This may help sustain the activation of the Ca^2+^ signaling system; however, the Ca^2+^ content is total Ca^2+^ rather than cytosolic Ca^2+^ dynamics, so this interpretation remains to be tested. Modareszadeh et al. [72] demonstrated that overexpression of *CAX3* enhances *SOS1* expression and antioxidant enzyme activities by elevating Ca^2+^ levels, thereby reducing Na^+^ accumulation and oxidative damage. Bashir et al. [73] reported that Fe, Zn, Mn, and Cu are essential cofactors for antioxidant enzymes, and their homeostasis is crucial for photosynthesis and reactive oxygen species (ROS) scavenging. In this study, OE-1 maintained higher levels of Fe, Zn, Mn, and Cu during the late stage of stress, which coincided with its higher SOD, POD, and CAT activities. These changes are consistent with the possibility that sustained trace-element availability contributes to the maintenance of antioxidant defense in OE-1 under stress; whether *SiFLA6* directly regulates ion homeostasis or antioxidant capacity remains to be investigated.

In this study, *FLA* family members were identified in foxtail millet, green foxtail, switchgrass, and sorghum. *FLA* genes contain various cis-acting elements associated with abiotic stress responses, and multiple *SiFLAs* are induced under various abiotic stress conditions. However, this needs to be verified by further qPCR experiments. SiFLA6 is located in the endoplasmic reticulum, but this needs to be further confirmed through the endoplasmic reticulum marker. *SiFLA6* is involved in the early response to saline–alkali stress. These findings suggest that *FLA* genes are important candidate genes involved in abiotic stress responses and serve as valuable genetic resources for marker-assisted breeding. Future studies should focus on functional validation and elucidation of the molecular mechanisms of *FLA* genes in multiple species to establish their regulatory networks.

## 4. Materials and Methods

### 4.1. Materials

In this study, the saline–alkali-tolerant foxtail millet cultivar ‘Jikegu 3’ (JK3) [74], previously identified by our group, and wild-type *Arabidopsis thaliana* (Columbia-0, Col-0) seeds were used as plant materials. The eukaryotic expression vector 1302-2Myc-eGFP was maintained in our laboratory. *Escherichia coli* DH5α and *Agrobacterium tumefaciens* GV3101 competent cells were purchased from Sangon Biotech (Shanghai) Co., Ltd. (Shanghai, China).

### 4.2. Identification of FLA Gene Family Members and Analysis of Protein Physicochemical Properties

Genomic data and annotation files for foxtail millet, switchgrass, sorghum, green foxtail, wheat, maize, and rice were retrieved from the Phytozome database. Protein sequences for these seven species were then extracted using TBtools. In addition, protein sequences of the *Arabidopsis* FLA gene family were obtained from the NCBI database (National Center for Biotechnology Information (nih.gov)) and used as queries for BLAST searches against the protein sequences of foxtail millet, green foxtail, switchgrass, and sorghum using TBtools (v2.476). The Hidden Markov Model (HMM) profile for the conserved FLA domain (Accession No. PF02469) was obtained from the Pfam database (http://pfam.xfam.org/, accessed on 23 August 2024). Candidate *FLA* genes in the four species were identified using Simple HMM Search. The overlap of the candidate genes identified by BLAST and HMM was used to determine the putative *FLA* gene family members. To validate the candidates, the protein sequences were subjected to conserved domain analysis using the SMART and the InterPro database. Sequences lacking complete conserved domains were discarded, and the final set of *FLA* gene family members in the four species was determined.

The physicochemical properties of all *FLA* family members, including molecular weight, isoelectric point, and hydrophobicity, were estimated using the Protein Parameter Calc tool in TBtools. Signal peptides were predicted using SignalP 4.1. Transmembrane regions were analyzed using TMHMM. In addition, the subcellular localization of all *FLA* family members was predicted using WoLF PSORT.

### 4.3. Phylogenetic Tree, Conserved Motifs, and Gene Structure Analysis of the FLA Gene Family

To investigate the phylogenetic relationships and classify the *FLA* genes, the FLA protein sequences of *Setaria italica*, *Setaria viridis*, *Panicum virgatum*, *Sorghum bicolor*, and *Arabidopsis thaliana* were aligned and subjected to phylogenetic analysis using the neighbour-joining (NJ) method in MEGA 11. The phylogenetic tree was further visualized using the online tool Interactive Tree of Life (iTOL) (https://itol.embl.de/). Based on their chromosomal positions, the identified genes were designated as *PvFLA1–46*, *SbFLA1–26*, *SiFLA1–24*, and *SvFLA1–25*, respectively.

Conserved motifs were analyzed using the MEME Suite Wrapper in TBtools, with the maximum number of motifs set to 10. Gene structures were analyzed using the Gene Structure View plugin. The hitdata file for the *FLA* gene family was obtained using the Batch Web CD-Search tool of the NCBI database. Finally, the distribution of gene structures, conserved domains, and motifs of all *FLA* family members was visualized using the Gene Structure View plugin in TBtools.

### 4.4. Chromosomal Localization, Duplication Events, and Synteny Analysis of the FLA Gene Family

Chromosomal locations of the *FLA* family members were obtained based on the genome annotation files of foxtail millet, green foxtail, switchgrass, and sorghum. Using the results of self-genome sequence alignment, the intraspecific evolutionary relationships among foxtail millet, green foxtail, *Arabidopsis*, and sorghum were visualized with Advanced Circos, and the tandem and segmental duplication events of their *FLA* genes were analyzed. Nonsynonymous (Ka) and synonymous (Ks) substitution rates were calculated using DnaSP V5.0, and selective pressure was assessed based on the Ka/Ks ratios: (1) Ka/Ks > 1 indicates positive selection; (2) Ka/Ks < 1 indicates purifying selection; and (3) Ka/Ks = 1 indicates neutral selection. Genomic and annotation data for all species except *Arabidopsis* were obtained from the Phytozome database. Furthermore, the One Step MCScanX tool was used to align the genome sequences of foxtail millet, green foxtail, switchgrass, sorghum, maize, rice, and *Arabidopsis*, thereby revealing the syntenic relationships among them.

### 4.5. Gene Gain and Loss, and Cis-Acting Element Analysis of the FLA Gene Family

The protein sequences of the *FLA* gene family from foxtail millet, switchgrass, green foxtail, sorghum, maize, rice, wheat, and *Arabidopsis* were submitted to the online TimeTree (http://timetree.org/home) tool to retrieve the species tree for the *FLA* family. Subsequently, the Gene Gain and Loss Analysis plugin in TBtools was used to analyze the expansion and contraction of *FLA* genes during evolution.

The upstream 2000 bp sequences of foxtail millet, switchgrass, sorghum, and green foxtail were submitted to the PlantCARE database (http://bioinformatics.psb.ugent.be/webtools/plantcare/html/) (accessed on 23 August 2024) to identify cis-acting elements in the promoter regions.

### 4.6. Expression Levels of SiFLAs Under Abiotic Stress and GO Enrichment Analysis

Transcriptomic data for the saline–alkali-tolerant cultivar ‘Jikegu 3’ (JK3) and the saline–alkali-sensitive line ‘Bao 175’ (B175) under saline–alkali stress (0, 12, and 24 h), previously obtained by our research group, were retrieved from the GSE278652 dataset [74]. Additional transcriptomic datasets for foxtail millet under diverse abiotic stresses were obtained from the NCBI database, including those for heat stress (PRJNA756390), drought stress (PRJEB21225), shading stress (PRJNA772942), and herbicide stress (PRJNA751769). Finally, heatmaps of expression profiles of *SiFLAs* in response to abiotic stress were constructed using TBtools v2.127.

The protein and genome sequences downloaded from the genomic database were used to generate a background GO annotation file for mRNA. Subsequently, GO enrichment analysis was carried out based on the gene IDs and the background annotation file to annotate gene functions. Finally, a histogram of the top 20 enriched GO terms was generated based on the *p*-values from the functional enrichment results.

### 4.7. Gene Cloning and Sequence Alignment of SiFLA6

Using JK3 as the template, the target gene was amplified via a two-step PCR strategy. In the first step, the gene was amplified using primers (*SiFLA6-1*) designed in the non-coding region. In the second step, the first-round PCR product served as the template for amplification with primers (*SiFLA6-2*) designed in the CDS region and harboring adapter sequences, yielding the target gene fragment (primer sequences are listed in Supplementary Table S4). The eukaryotic expression vector 1302-2Myc-eGFP (CaMV 35S promoter) was linearized with the BstBI restriction enzyme. The linearized vector was gel-purified and then ligated to the target gene fragment using seamless cloning with the 2.5× OK Clon Master Mix (Accurate Biotechnology Co., Ltd.) (Changsha, China). The ligation products were transformed into DH5α competent cells. The transformed cells were plated and incubated inverted at 37 °C for 12–16 h. Single colonies were then picked and cultured in liquid medium, and the resulting cultures were used directly as templates for colony PCR verification. Positive clones that yielded the expected band size were sent to Sangon Biotech (Shanghai) Co., Ltd. for sequencing to confirm the correct sequence.

Based on the SiFLA6 protein sequence, 10 homologous proteins were identified by BLASTp searches against the NCBI database (National Center for Biotechnology Information (nih.gov)), with the following accession numbers: XP_051186444.1, XBI80494.1, CAN8507514.1, KAL6842475.1, KAG2583123.1, KAK3118831.1, XP_062200000.1, PWZ13922.1, WVZ64689.1, and RLM69277.1. Multiple sequence alignment of these homologous proteins was conducted using DNAMAN software, and a phylogenetic tree was constructed using the neighbor-joining method in MEGA 11.

### 4.8. Generation of SiFLA6-Overexpressing Plants and Subcellular Localization

The recombinant eukaryotic expression plasmid 1302-2Myc-eGFP-*SiFLA6* was introduced into *Agrobacterium tumefaciens* GV3101 competent cells by the freeze–thaw method. Wild-type (WT) seeds were sown onto solid MS medium, stratified at 4 °C in the dark for 3 days, and then transferred to a growth chamber under a photoperiod of 16 h light/8 h dark at 22 ± 2 °C with a light intensity of 180 µmol/m^2^/s. When the seedlings had developed 4 to 6 true leaves, they were transferred to a nutrient soil mixture (nutrient soil: vermiculite = 3: 2) and grown until the onset of bolting. The recombinant plasmid was introduced into *Agrobacterium tumefaciens* GV3101, and the resulting strain was then used to transform wild-type *Arabidopsis* by the floral dip method. The T_1_ seeds were harvested from mature transgenic plants and screened with 30 mg/L hygromycin B. Surviving seedlings were transplanted into soil, and leaf samples were collected for PCR identification using two primer sets. The target-gene primer *SiFLA6-2* was expected to amplify a 1249 bp fragment, consisting of a 1212 bp target gene and a 37 bp adaptor sequence, with an annealing temperature of 58 °C. The sequencing primers for the 1302-2Myc-eGFP vector had an annealing temperature of 55 °C (Supplementary Table S4). T_2_ seeds harvested from PCR-positive T_1_ plants were subjected to hygromycin screening. T_2_ populations showing a 3:1 resistance segregation ratio, and resistant seedlings were transplanted. T_3_ seeds from these T_2_ plants were further screened to obtain non-segregating T_3_ lines. Finally, homozygous overexpression lines of *SiFLA6* (OE-1 and OE-2) were generated for subsequent salinity-alkalinity stress assays (Supplementary Figure S5).

The OE-1 plants were grown to the vegetative stage. Fresh leaves of uniform vigor and size were collected, and the mid-sections were cut into small pieces to prepare slides for microscopic observation. Slides were examined using a laser scanning confocal microscope (FV3000, Olympus Corporation, Tokyo, Japan). The subcellular localization of *SiFLA6* was analyzed by detecting the eGFP fluorescence signal.

### 4.9. Growth, Physiological, and Biochemical Analyses of SiFLA6-Overexpressing Arabidopsis Under Saline–Alkali Stress

Germination stress experiment: Solid MS media supplemented with 0%, 10%, 20%, 30%, and 40% seawater were prepared using filter-sterilized seawater (collected from the coast of Haigang District, Qinhuangdao, Bohai Sea, China). The corresponding salinities were approximately 2.46‰, 3.78‰, 5.36‰, and 6.92‰, with pH values of 7.74, 7.82, 7.88, and 7.92, respectively. Uniform seeds of WT, OE-1, and OE-2 were selected and sown on solid MS media containing different concentrations of seawater. The inoculated plates were placed in a constant-temperature incubator and cultured under uniform conditions (16 h light/8 h dark photoperiod, 22 ± 2 °C, and 80% light intensity). After 10 days of treatment, the growth phenotypes of the seedlings under each seawater concentration were observed and photographed.

Seedling stress experiment: Seeds of WT and OE-1 were sown in pots containing vermiculite and watered with Hoagland nutrient solution. The plants were grown in a growth chamber for approximately 2.5 weeks (under the same conditions as described above). Subsequently, saline–alkali stress was applied using the 50% seawater concentration (salinity: 8.8‰, pH: 7.96) (Supplementary Figure S6). During the treatment, the growth status of the plants was observed and recorded at 0, 24, 48, 72, 96, and 120 h after stress treatment. Additionally, plant leaves were collected at each time point for the measurement of various indicators. The measured morphological parameters included the fresh and dry weights of whole plants and the leaf SPAD value. For physiological assays related to redox homeostasis, above-ground tissues were used. Specifically, the contents of malondialdehyde (MDA), hydrogen peroxide (H_2_O_2_), and proline (Pro) were determined using the thiobarbituric acid method, the titanium sulfate colorimetric method, and the sulfosalicylic acid extraction method, respectively; whereas the activities of superoxide dismutase (SOD), peroxidase (POD), and catalase (CAT) were measured using the WST-8 method, the guaiacol oxidation method, and the ammonium molybdate method, respectively. All assays were performed with three biological replicates per treatment. Intact whole plants were collected and killed at 105 °C for 30 min. The samples were then oven-dried at 75 °C to a constant weight, ground into powder, and passed through a sieve. Sample digestion was performed using the H_2_SO_4_-H_2_O_2_ method. The contents of Na^+^ and K^+^ were determined by flame photometry, while the contents of Ca^2+^, Mg^2+^, Fe, Cu, Mn, and Zn were measured using inductively coupled plasma (ICP) spectrometry. Total RNA was extracted and purified using the SteadyPure Plant RNA Extraction Kit. The purified RNA was reverse-transcribed into cDNA with the Evo M-MLV Reverse Transcription Premix Kit. Quantitative real-time PCR (qRT-PCR) was carried out using the SYBR Green Pro Taq HS Premix qPCR Kit with gene-specific primers for *SiFLA6* (*SiFLA6-3*) and the *Arabidopsis* reference gene *Actin2* (primer sequences are listed in Supplementary Table S4). All kits were purchased from Accurate Biotechnology Co., Ltd. For each sample, three biological replicates and three technical replicates were set up. Finally, the relative expression level of *SiFLA6* in different lines was calculated using the 2^−ΔΔC^_t_ method.

### 4.10. Data Statistics

Data statistical analysis was performed using Excel 2021 and DPS 9.01. One-way analysis of variance (ANOVA) combined with the least significant difference (LSD) test was used to detect intergroup differences. Statistical significance was set at *p* < 0.05, and results were expressed as mean ± standard deviation (SD). Figures and visualizations were generated and polished with Excel, TBtools and Adobe Illustrator 2023.

## 5. Conclusions

In this study, a total of 121 *FLA* family members were identified and classified into four subfamilies in four Poaceae species. The evolution of the *FLA* family was primarily driven by purifying selection. Furthermore, *FLA* genes contain various *cis*-acting elements associated with abiotic stress responses. Under seawater treatment, the OE-1 line exhibited higher K^+^/Na^+^ and Ca^2+^/Na^+^ ratios, reduced Na^+^ accumulation with relatively preserved K+ accumulation, and maintained higher Ca^2+^, Mg^2+^, Cu, Fe, Zn and Mn concentrations than the wild type (particularly from 48 h onward). Additionally, the OE-1 line enhanced SOD, POD, and CAT activities, promoted proline (Pro) accumulation, and reduced MDA and H_2_O_2_ contents. Collectively, these findings indicate that the ion and redox balance in OE-1 is less disrupted, implying that *SiFLA6* is a positive regulator for *Arabidopsis* in response to saline–alkali tolerance.

## Data Availability

Data will be made available on request.

## Supplementary Materials

The following supporting information can be downloaded at https://www.mdpi.com/article/10.3390/plants15213260/s1. Supplementary Table S1. Analysis of the physicochemical properties of FLA proteins. Supplementary Table S2. Calculation of the Ka/Ks ratios of duplicated gene pairs in the *FLA* gene family of foxtail millet, green foxtail, switchgrass and sorghum. Supplementary Table S3. Collinear orthologous gene pairs of FLA family between *Setaria italica* and six other species. Supplementary Table S4. Specific primer sequences for *SiFLA6* gene cloning and qRT-PCR. Supplementary Figure S1. Gene structure and conserved motifs of the *FLA* gene family in foxtail millet, green foxtail, switchgrass and sorghum. Supplementary Figure S2. Gene duplication analysis of the *FLA* gene family in foxtail millet, green foxtail, switchgrass, and sorghum. (A) Gene duplication of *SiFLAs* and *SvFLAs*. (B) Gene duplication of *PvFLAs* and *SbFLAs*. Supplementary Figure S3. Analysis of *cis*-acting elements in the *FLA* gene family of foxtail millet, green foxtail, switchgrass, and sorghum. Supplementary Figure S4. Cloning of the *SiFLA6* gene and construction of its expression vector. (A) Target gene amplification of *SiFLA6*. (B) Bacterial liquid PCR of *SiFLA6*. The numbers 1–6 indicate the length of the target gene fragment. M: Marker DL5000. Supplementary Figure S5. Screening of homozygous positive plants of *SiFLA6*. (A) T_1_ positive seedlings identified by screening. (B,C) T_2_ positive seedlings identified by screening, including individual plant 4 (OE-1) and individual plant 22 (OE-2), respectively. (D,E) T_3_ homozygous positive seedlings identified by screening, including OE-1 and OE-2, respectively. (F) PCR identification of T_1_ positive seedlings. 1–2: WT with primer 1, WT with primer 2; 3–4: individual plant 4 (OE-1) with primer 1, individual plant 4 (OE-1) with primer 2; 5–6: individual plant 15 with primer 1, individual plant 15 with primer 2; 7–8: individual plant 22 (OE-2) with primer 1, individual plant 22 (OE-2) with primer 2. Primer 1 targets SiFLA6, and Primer 2 is the universal sequencing primer of the 1302 vector. Supplementary Figure S6. Screening of the seawater treatment concentration in wild-type *Arabidopsis*. (A–C) Relative chlorophyll content (SPAD value) (A), dry weight (B) and fresh weight (C) of wild-type *Arabidopsis* seedlings after 120 h of treatment with 0, 25, 50, 75 and 100% seawater. Data are means ± SD. Different letters above bars indicate significant differences at *p* < 0.05 (one-way ANOVA followed by Tukey’s HSD test); bars sharing the same letter are not significantly different.
