# Supplementary material for: Analysis of FLA Gene Family in Four Poaceae Species and Functional Characterization of SiFLA6 in Saline–Alkali Tolerance
Source: Plants (Basel). 2026 Oct 22;15(21):3260. doi: 10.3390/plants15213260 (PMC13765344; doi:10.3390/plants15213260)
Supplement: Supplementary file 1 [file plants-15-03260-s001.zip › Supplementary Figure S1.pdf]

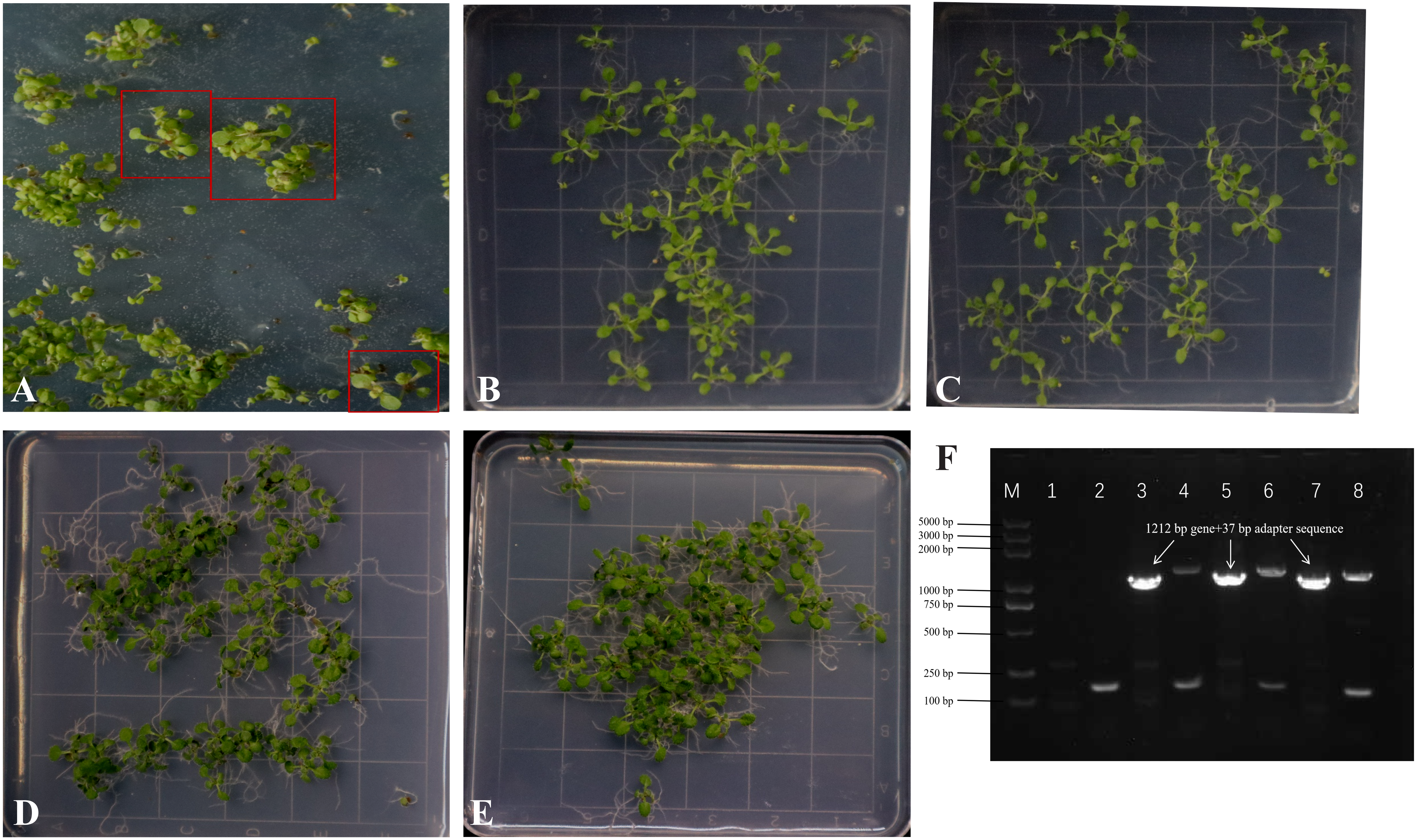
A
B
C
D
E
F
M 1 2 3 4 5 6 7 8
5000 bp
3000 bp
2000 bp
1000 bp
750 bp
500 bp
250 bp
100 bp
1212 bp gene+37 bp adapter sequence
