# Supplementary material for: Analysis of FLA Gene Family in Four Poaceae Species and Functional Characterization of SiFLA6 in Saline–Alkali Tolerance
Source: Plants (Basel). 2026 Oct 22;15(21):3260. doi: 10.3390/plants15213260 (PMC13765344; doi:10.3390/plants15213260)
Supplement: Supplementary file 1 [file plants-15-03260-s001.zip › Supplementary Figure S2.pdf]

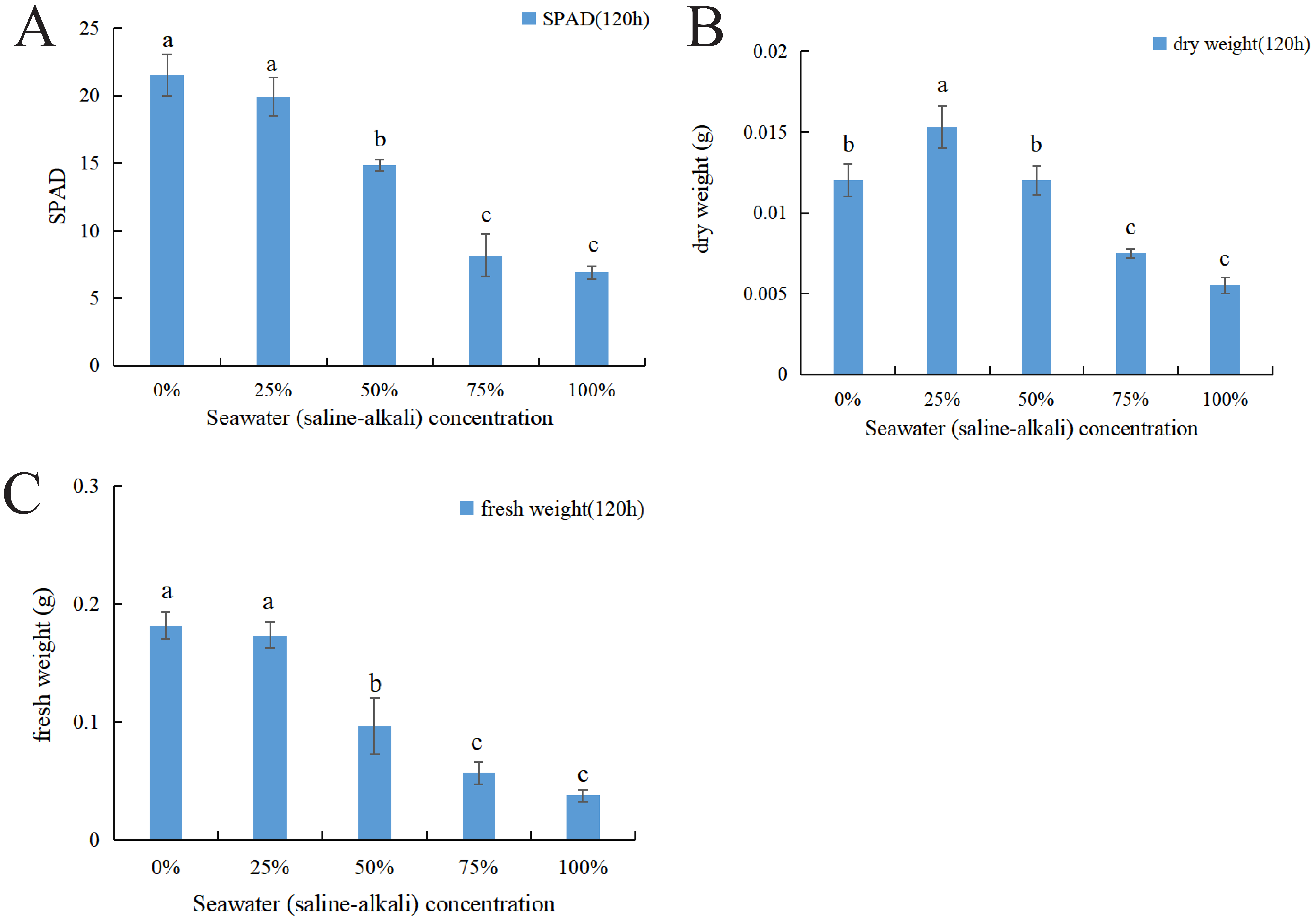
A
SPAD(120h)
SPAD
25
20
15
10
5
0
a
a
b
c
c
0%
25%
50%
75%
100%
Seawater (saline-alkali) concentration
B
dry weight(120h)
dry weight (g)
0.02
0.015
0.01
0.005
0
b
a
b
c
c
0%
25%
50%
75%
100%
Seawater (saline-alkali) concentration
C
fresh weight(120h)
fresh weight (g)
0.3
0.2
0.1
0
a
a
b
c
c
0%
25%
50%
75%
100%
Seawater (saline-alkali) concentration
