# Supplementary material for: Analysis of FLA Gene Family in Four Poaceae Species and Functional Characterization of SiFLA6 in Saline–Alkali Tolerance
Source: Plants (Basel). 2026 Oct 22;15(21):3260. doi: 10.3390/plants15213260 (PMC13765344; doi:10.3390/plants15213260)
Supplement: Supplementary file 1 [file plants-15-03260-s001.zip › Supplementary Figure S3.pdf]

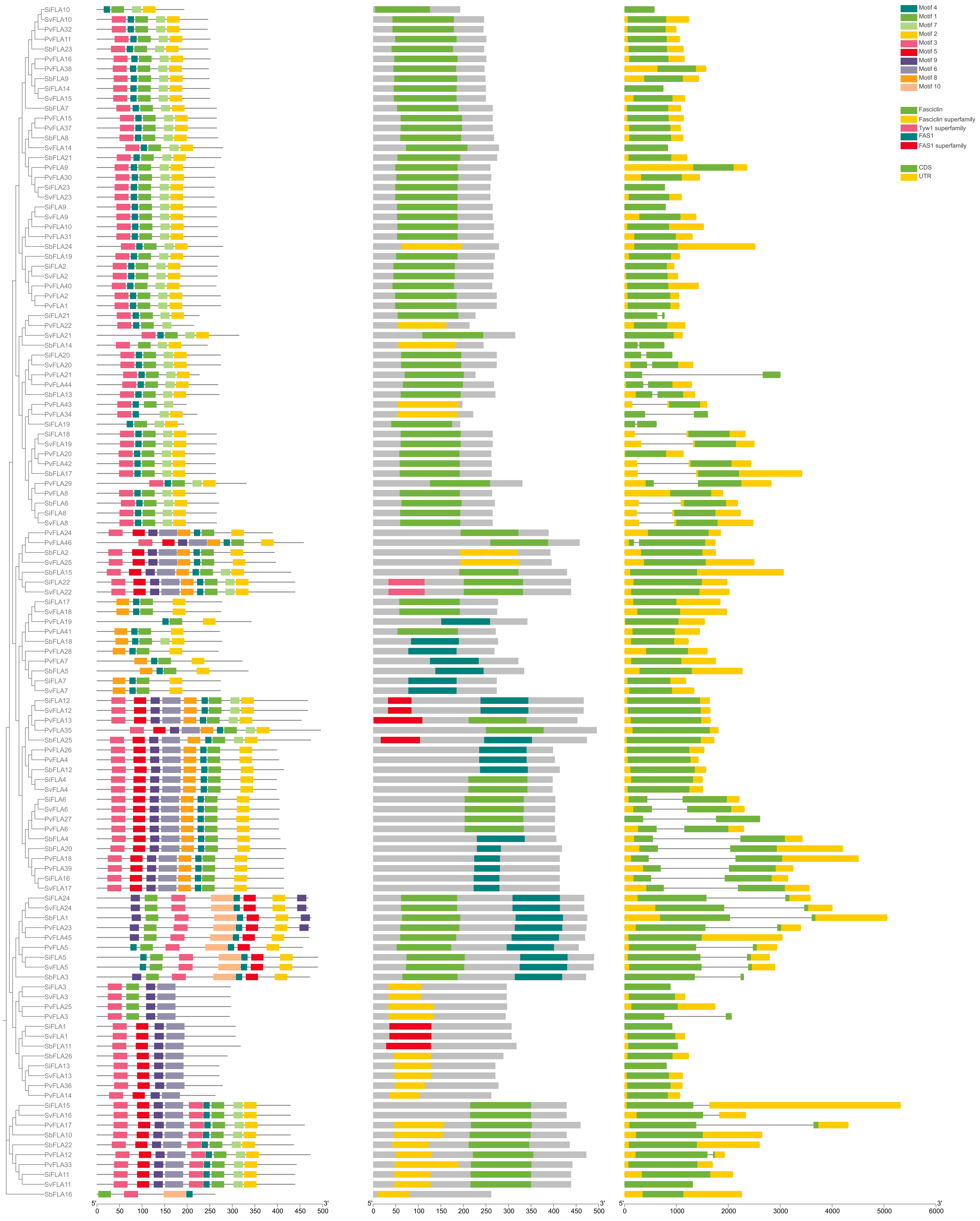

SiFLA10
SvFLA10
PvFLA32
PvFLA11
SbFLA23
PvFLA16
PvFLA38
SbFLA9
SiFLA14
SvFLA15
SbFLA7
PvFLA15
PvFLA37
SbFLA8
SvFLA14
SbFLA21
PvFLA9
PvFLA30
SiFLA23
SvFLA23
SiFLA9
SvFLA9
PvFLA10
PvFLA31
SbFLA24
SbFLA19
SiFLA2
SvFLA2
PvFLA40
PvFLA2
PvFLA1
SiFLA21
PvFLA22
SvFLA21
SbFLA14
SiFLA20
SvFLA20
PvFLA21
PvFLA44
SbFLA13
PvFLA43
PvFLA34
SiFLA19
SiFLA18
SvFLA19
PvFLA20
PvFLA42
SbFLA17
PvFLA29
PvFLA8
SbFLA6
SiFLA8
SvFLA8
PvFLA24
PvFLA46
SbFLA2
SvFLA25
SbFLA15
SiFLA22
SvFLA22
SiFLA17
SvFLA18
PvFLA19
PvFLA41
SbFLA18
PvFLA28
PvFLA7
SbFLA5
SiFLA7
SvFLA7
SiFLA12
SvFLA12
PvFLA13
PvFLA35
SbFLA25
PvFLA26
PvFLA4
SbFLA12
SiFLA4
SvFLA4
SiFLA6
SvFLA6
PvFLA27
PvFLA6
SbFLA4
SbFLA20
PvFLA18
PvFLA39
SiFLA16
SvFLA17
SiFLA24
SvFLA24
SbFLA1
PvFLA23
PvFLA45
PvFLA5
SiFLA5
SvFLA5
SbFLA3
SiFLA3
SvFLA3
PvFLA25
PvFLA3
SiFLA1
SvFLA1
SbFLA11
SbFLA26
SiFLA13
SvFLA13
PvFLA36
PvFLA14
SiFLA15
SvFLA16
PvFLA17
SbFLA10
SbFLA22
PvFLA12
PvFLA33
SiFLA11
SvFLA11
SbFLA16
5' 0 50 100 150 200 250 300 350 400 450 500 3'
5' 0 50 100 150 200 250 300 350 400 450 500 3'
5' 0 1000 2000 3000 4000 5000 6000 3'
Motif 4
Motif 1
Motif 7
Motif 2
Motif 3
Motif 5
Motif 9
Motif 6
Motif 8
Motif 10
Fasciclin
Fasciclin superfamily
Tyw1 superfamily
FAS1
FAS1 superfamily
CDS
UTR
