# Supplementary material for: Analysis of FLA Gene Family in Four Poaceae Species and Functional Characterization of SiFLA6 in Saline–Alkali Tolerance
Source: Plants (Basel). 2026 Oct 22;15(21):3260. doi: 10.3390/plants15213260 (PMC13765344; doi:10.3390/plants15213260)
Supplement: Supplementary file 1 [file plants-15-03260-s001.zip › Supplementary Figure S4.pdf]

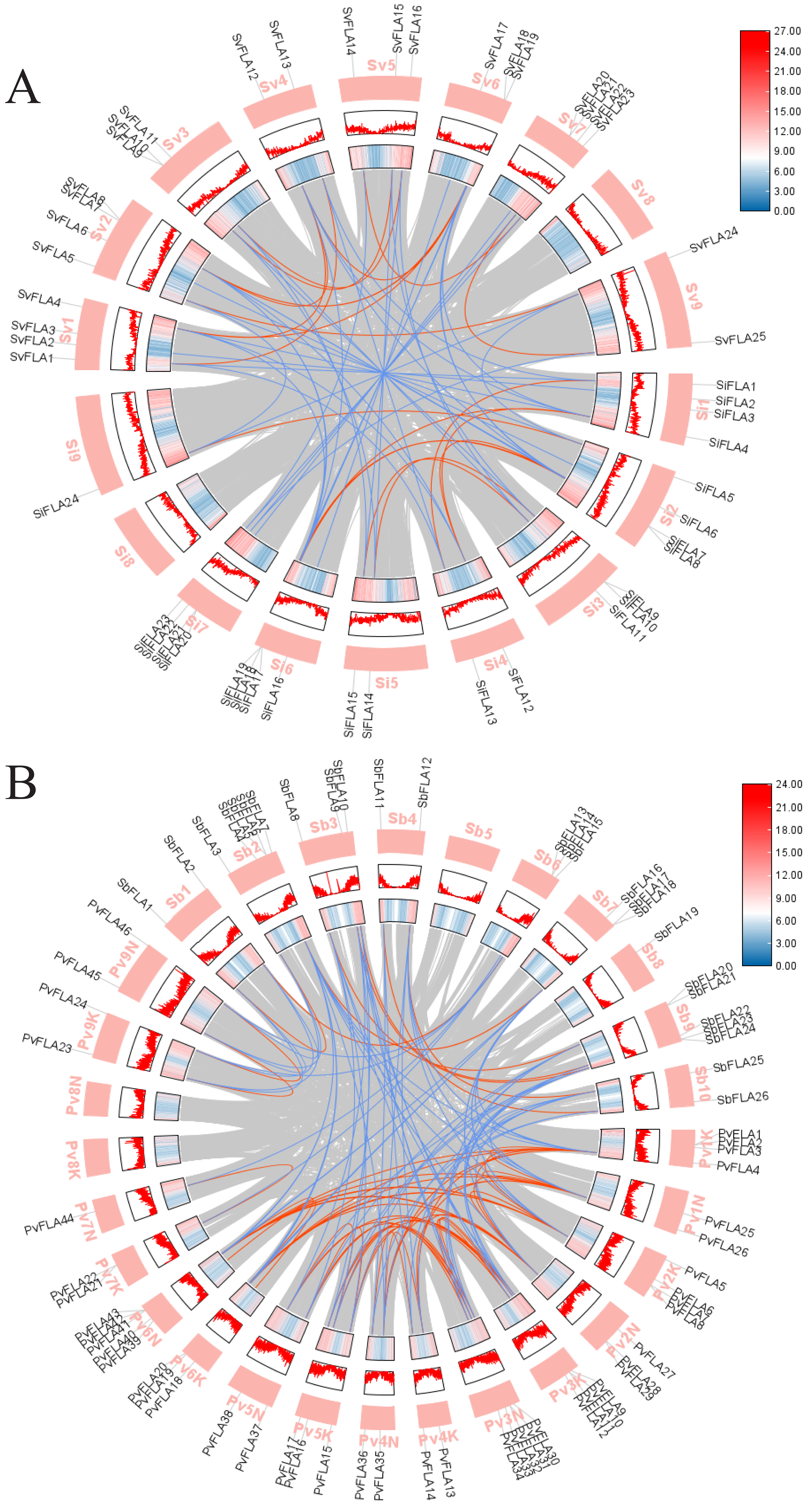

A
27.00
24.00
21.00
18.00
15.00
12.00
9.00
6.00
3.00
0.00
Sv1
Sv2
Sv3
Sv4
Sv5
Sv6
Sv7
Sv8
Sv9
Si1
Si2
Si3
Si4
Si5
Si6
Si7
Si8
Si9
B
24.00
21.00
18.00
15.00
12.00
9.00
6.00
3.00
0.00
Sb1
Sb2
Sb3
Sb4
Sb5
Sb6
Sb7
Sb8
Sb9
Sb10
Pv1K
Pv1N
Pv2K
Pv2N
Pv3K
Pv3N
Pv4K
Pv4N
Pv5K
Pv5N
Pv6K
Pv6N
Pv7K
Pv7N
Pv8K
Pv8N
Pv9K
Pv9N
