# Supplementary material for: Analysis of FLA Gene Family in Four Poaceae Species and Functional Characterization of SiFLA6 in Saline–Alkali Tolerance
Source: Plants (Basel). 2026 Oct 22;15(21):3260. doi: 10.3390/plants15213260 (PMC13765344; doi:10.3390/plants15213260)
Supplement: Supplementary file 1 [file plants-15-03260-s001.zip › Supplementary Figure S6.pdf]

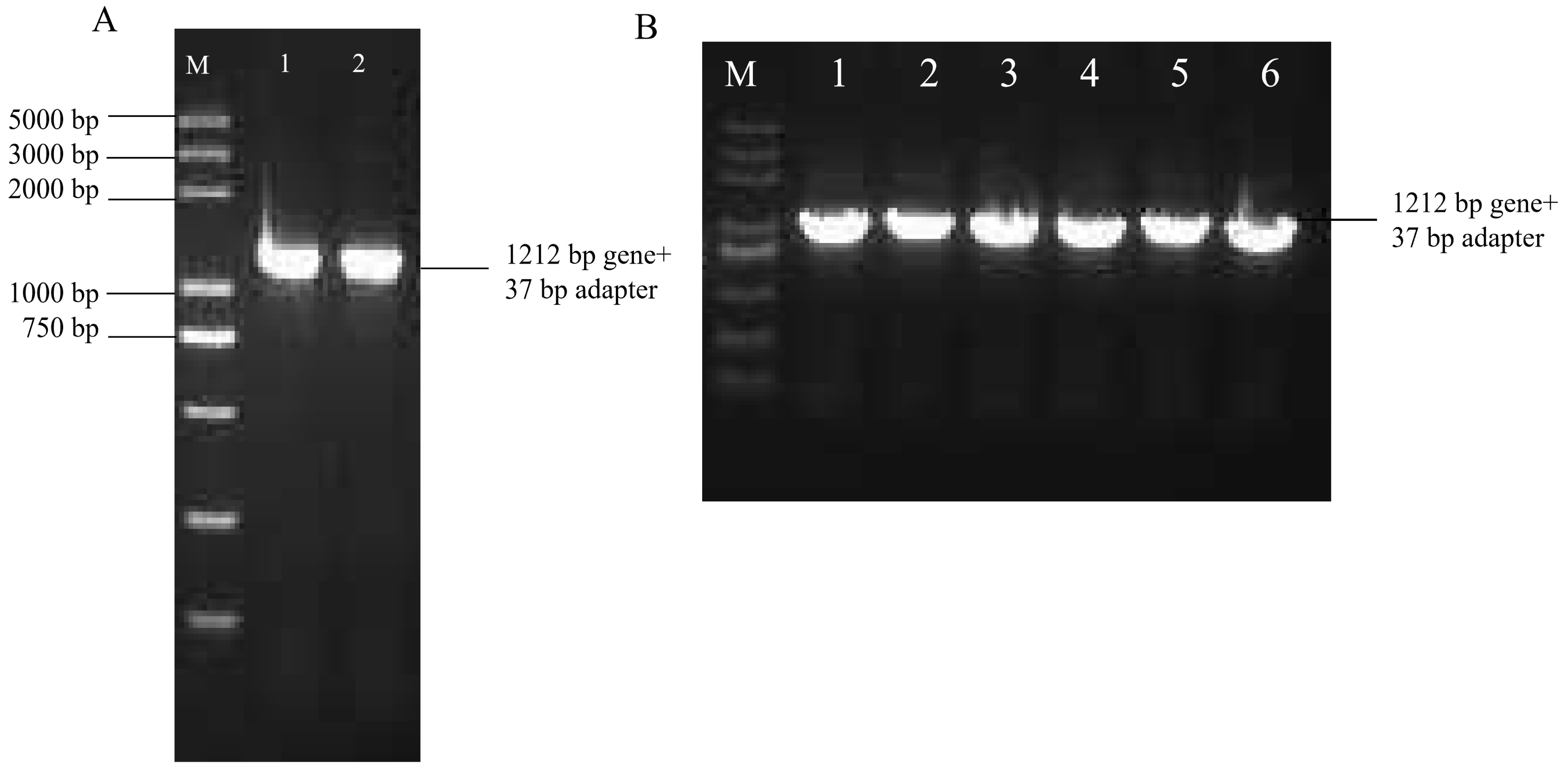
A
M
1
2
5000 bp
3000 bp
2000 bp
1000 bp
750 bp
1212 bp gene+
37 bp adapter
B
M
1
2
3
4
5
6
1212 bp gene+
37 bp adapter
